# Genetic association and causal inference between lung function and venous thromboembolism

**DOI:** 10.1186/s12931-023-02335-3

**Published:** 2023-01-30

**Authors:** Qiaoyun Zhang, Xiaoyu Zhang, Jie Zhang, Mengyang Jiang, Yiqiang Zhang, Deqiang Zheng, Lijuan Wu, Wei Wang, Baoguo Wang, Youxin Wang

**Affiliations:** 1 Beijing Key Laboratory of Clinical Epidemiology, School of Public Health, Capital Medical University, No. 10 Xitoutiao, Youanmenwai Street, Fengtai District, Beijing, 100069 China; 2 Department of Anesthesiology, Beijing Sanbo Brain Hospital, Capital Medical University, 50 Yikesong Road, Haidian District, Beijing, 100093 China; 3 Centre for Precision Medicine, Edith Cowan University, Joondalup, WA Australia

**Keywords:** Forced expiratory volume in one second, Forced vital capacity, Peak expiratory flow, Venous thromboembolism, Deep vein thrombosis, Pulmonary embolism, Mendelian randomization, Genetic correlation

## Abstract

**Background:**

Previous studies have indicated that lower lung function is related to a higher risk of venous thromboembolism (VTE). However, causal inferences may be affected by confounders, coheritability or reverse causality. We aimed to explore the causal association between lung function and VTE.

**Methods:**

Summary data from public genome-wide association studies (GWAS) for lung function and VTE were obtained from published meta-analysis studies and the FinnGen consortium, respectively. Independent genetic variables significantly related to exposure were filtered as proxy instruments. We adopted linkage disequilibrium score regression (LDSC) and two-sample Mendelian randomization (MR) analyses to infer the genetic backgrounds and causal associations between different lung functions and VTE events.

**Results:**

LDSC showed a genetic correlation between forced expiratory volume in one second (FEV1) and deep vein thrombosis (DVT) (rg = − 0.189, *P* = 0.005). In univariate MR (UVMR), there was suggestive evidence for causal associations of genetically predicted force vital capacity (FVC) with DVT (odds ratio (OR) 0.774; 95% confidence interval (CI) 0.641–0.934) via forwards analysis and genetically predicted pulmonary embolism (PE) with FVC (OR 0.989; 95% CI 0.979–0.999) via reverse analysis. Multivariate MR (MVMR) analyses of lung function-specific SNPs suggested no significant direct effects of lung function on VTE, and vice versa. Of note is the borderline causal effect of PE on FEV1 (OR 0.921; 95% CI 0.848–1.000).

**Conclusions:**

Our findings identified a coheritability of FEV1 (significant) and FVC (suggestive) with DVT. There was no convincing causal relationship between lung function and the risk of VTE events. The borderline causal effect of PE on FEV1 and the significant genetic correlation of FEV1 with DVT may have clinical implications for improving the quality of existing prevention and intervention strategies.

**Supplementary Information:**

The online version contains supplementary material available at 10.1186/s12931-023-02335-3.

## Background

Venous thromboembolism (VTE) is a chronic disease manifested as either deep vein thrombosis (DVT) or as pulmonary embolism (PE) according to the site of embolism and is the third leading cause of vascular mortality, affecting nearly 10 million people worldwide each year [1, 2]. VTE is a multicausal disorder influenced by both acquired and inherited risk factors and is related to reduced survival and high recurrence rates and health-care costs [3–5]. Previous studies have focused on clinical risk factors (cancer, major surgery, immobilization, etc.) and some specific genetic conditions (i.e., Factor V, protein C or protein S) account for less than one-fifth of the population attributable risk in the elderly [6, 7], but most VTEs are provoked by weak risk factors or even no identifiable risk factors [1, 8]. Published evidence indicates that poor lung function is related to increased atherothrombotic risk, with risk factors broadly similar to those for VTE [9, 10]. Moreover, chronic lung diseases (e.g., chronic obstructive pulmonary disease (COPD) [9, 11], asthma, emphysema, interstitial lung disease [12]) or postoperative lung cancer [13], characterized by varying degrees of impairment in different lung function parameters, are related to an increased risk of VTE [10]. Another study pointed out that impaired lung function did not affect the VTE risk in cancer patients complicated with PE [14].

Forced expiratory volume in one second (FEV1), forced vital capacity (FVC), the ratio of FEV1 to FVC (FEV1/FVC) and peak expiratory flow (PEF) are key indicators for monitoring lung function and can also predict the morbidity and mortality of different respiratory diseases [15–17] as well as all-cause mortality [18]. A retrospective observational study indicated that the more severe the airway obstruction in COPD patients, the higher the risk of VTE [12], and another autopsy study noted that patients with emphysema were at higher risk of VTE [19]. However, no studies have investigated whether different parameters of lung function are causally associated with VTE events.

Based on the aforementioned inconsistent conclusions, the lack of public awareness that unprovoked thrombus is common and preventable as well as the increasing prevalence of impaired lung function and the serious impact on quality of life [20], clarifying the underlying causality and the direction of these relations would be conducive to guiding management and prevention. In addition, the intercorrelations among different parameters of lung function and the potential shared risk factors (e.g., age, sex [11, 21, 22]) of lung function and VTE events pose a great challenge to clarify the causal relation between lung function and VTE.

The design of the Mendelian randomization (MR) study follows Mendel’s law of inheritance, which is similar to a randomized controlled trial (RCT) and can overcome unmeasured confounders and provide more robust evidence for causal estimation between different lung functions and VTE events. Genetic variants significantly associated with lung function and VTE events were selected as proxy instrumental variables (IVs). IVs are less likely to be influenced by confounders and reverse causality as the random assignment of parents to offspring at conception [23, 24]. However, there is the possibility of pleiotropy, exemplified by a univariate MR (UVMR) analysis of FEV1 as an exposure factor. Figure S1 (Additional file 1) shows the possible explanations for the association of single nucleotide polymorphisms (SNPs) with FEV1 exposure and VTE outcomes. Vertical pleiotropy refers to the fact that SNPs robustly related to FEV1 affect VTE outcomes via other lung function traits first and then downstream affect FEV1 (Additional file 1: Fig. S1A). Horizontal pleiotropy means that SNPs related to FEV1 affect VTE outcomes via other lung function traits without mediation by FEV1 (Additional file 1: Fig. S1B). Confounding pleiotropy occurs when SNPs affect VTE outcomes via lung function parameters other than FEV1, even though SNPs affect FEV1 via other lung function parameters (Additional file 1: Fig. S1C). Multivariable MR (MVMR), taking into account the possible pleiotropy described above, integrates a set of pleiotropic SNPs [25] related to at least one exposure as IVs to assess the direct causal effect of each exposure on the outcomes (Additional file 1: Fig. S2). Moreover, in the case of horizontal pleiotropy, causality can be inferred even if no IV indicates a specific association with any independent exposure [26]. Linkage disequilibrium score (LDSC) regression was performed to investigate the coheritability between different lung function traits and VTE events by assessing the genetic correlation [27]. Here, we adopted bidirectional, UVMR and MVMR methods to infer the causal association of different lung function parameters with the risk of VTE and its subtypes (DVT and PE) using summary GWAS data from a European population.

## Methods

### Study design

This is a two-sample bidirectional, UVMR and MVMR study. The genetic variants significantly related to lung function parameters (FEV1, FVC, FEV1/FVC and PEF) and VTE events (VTE, DVT and PE) were selected as IVs, respectively. A schematic diagram of the study design is shown in Fig. S3 (Additional file 1). The valid IV should satisfy the following three core assumptions. First, the proxy IVs should be strongly correlated with exposure. Second, the proxy IVs had no associations with confounders. Last, IVs should only be linked with VTE via lung function. All statistical analyses in our study were based on publicly available summary data; therefore, no ethical approval was needed.

### GWAS of lung function (FEV1, FVC, FEV1/FVC and PEF)

Summary data for different lung functions were derived from the largest publicly available GWAS, which included 19,819,139 variants in 400,102 individuals of European ancestry (UK Biobank: 321,407 and SpiroMeta: 79,055). In each of the original GWASs, linear regression models were fitted for each trait (FEV1, FVC, FEV1/FVC and PEF) with age, age^2^, sex, height, smoking status (ever/never) and genotyping array as a series of covariates. All the residuals from linear regression were rank-based inverse-normal transformed to obtain normally distributed Z-scores, which were used as the phenotype for the association test. Details are available elsewhere [28], and summary statistics are available for download via LD-Hub (http://ldsc.broadinstitute.org/ldhub/).

### GWAS of VTE (VTE, DVT and PE)

We used summary statistics from a GWAS that was made public by the FinnGen consortium [29] (Release 5, https://r5.finngen.fi/), including 9176 cases and 209,616 controls for VTE (Phenocode: I9_VTE). Replication analyses were performed using 4576 cases and 214,216 controls for DVT of the lower extremities (no controls excluded) (Phenocode: I9_PHLETHROMBDVTLOW_EXNONE) and 4185 cases and 214,607 controls for pulmonary embolism (no controls excluded) (Phenocode: I9_PULMEMB_EXNONE). A total of 19,023 cases and 195,144 controls were included in the FinnGen consortium, and a total of 16,962,023 variants were analysed using a mixed-model logistic regression model with adjustment for age, sex, 10 principal components (PCs) and genotyping batch. VTE, DVT and PE were all defined according to the International Classification of Diseases (ICD) revision 9.

The GWAS summary data on exposure and outcome were all based on European populations.

### Genetic instrumental variable selection

The SNPs that were significantly (*P* threshold = 5 × 10^−8^) associated with lung function and VTE were selected as instrumental variables, respectively. Independent variants (r^2^ < 0.001, window size = 10,000 kb) were retained according to European ancestry reference data from the 1000 Genomes Project. The above procedures were performed with R (version 4.0.3) software.

### UVMR analyses

Forwards UVMR analyses were conducted to assess the causal relation between different lung function parameters and VTE events. Then, reverse UVMR was performed using genetic variants with VTE, DVT and PE to estimate their causal effects on different lung function parameters. The effects (i.e., beta) and the corresponding lung were obtained from the GWAS-lung function and GWAS-VTE [30]. IVs having direct effects on the outcome were excluded (*P* < 1 × 10^−5^), and palindromic SNPs were removed by harmonizing lung function and VTE data [31].

Inverse variance-weighted (IVW) analysis was performed as the main approach, which was actually a single variable weighted linear regression of outcome-SNP effects on exposure-SNP effects, and the intercept was constrained to zero [32]. The results may be imprecise if IVs exhibit horizontal pleiotropy, meaning that IVs may affect outcomes via pathways other than exposures [33]. Therefore, we supplementarily applied several MR methods based on different IV assumptions, including weighted median, weighted mode, MR-Egger regression and causal analysis using summary effect estimates (CAUSE) approaches, as sensitivity analyses to verify the robustness of the main IVW estimate [33]. The MR-Egger regression, of which the intercept is not constrained to zero [33, 34], gives consistent estimates with the IVW method if all IVs are invalid, while the weighted median method requires more than half of the IVs to be valid [35]. The weighted mode method is inherently robust to IVs with outlier ratio estimates and is not as susceptible to a small number of pleiotropic variants as the IVW and MR-Egger approaches [36]. For efficiency, weighted median estimates are generally as accurate as the IVW method, both are more accurate than MR-Egger regression, and MR-Egger regression is especially imprecise if IVs are all similarly associated with the exposure [35]. Horizontal pleiotropy may be correlated (IVs affect exposure and outcome through shared factors) or not correlated (IVs affect exposure and outcome via independent pathways) with a shared factor, but both do not violate the major MR assumption [37]. CAUSE analysis, a recent method that accounts for correlated or uncorrelated horizontal pleiotropy effects, was conducted, which includes more IVs by LD pruning (r^2^ < 0.1) with its built-in function based on precomputed LD estimates [37].

Horizontal pleiotropy was evaluated by the intercept test of the MR-Egger method (the intercept *P*_value < 0.05 implied the presence of horizontal pleiotropy) [38] and the MR pleiotropy residual sum and outlier (MR-PRESSO) test (potential outlier SNPs that violated the IV assumptions could be detected) [39]. In addition, heterogeneity was estimated by the Cochran *Q* test and *I*^2^ statistics in the IVW method (the Cochran *Q*_*P* value < 0.05 or *I*^2^ statistics > 25% indicated the presence of heterogeneity) as well as leave-one-out sensitivity analysis [40, 41], which could help to evaluate horizontal pleiotropy.

### MVMR analyses

Forwards MVMR analyses were conducted to infer the direct effects of each lung function parameter while accounting for the effects of the others. An extension of the IVW method (uncorrected variants, random effect model) to perform multivariate weighted linear regression [33] and an extension of the MR-Egger regression method to correct for both measured and unmeasured pleiotropy were conducted in MVMR [42]. Then, we repeated the MVMR method in the reverse analyses to estimate the direct effects of VTE, DVT or PE on different lung function parameters.

Odds ratios (ORs) and corresponding 95% confidence intervals (CIs) of lung function correspond to the risk of VTE events per standard deviation (SD) increase in log odds of different lung function parameters, and vice versa. Two-sided α < 0.007 (0.05/7, Bonferroni corrected significance threshold) was defined as a statistically significant difference since four potential risk factors and three outcomes were included in our study. *P* < 0.05 but above 0.007 was defined as suggestive evidence for a potential association. MR analyses were conducted using the following R (version 4.0.3) packages: “TwoSampleMR”, “MR-PRESSO” and “CAUSE”.

### Variance explained by IVs and F_statistic of MR analyses

To estimate the variance explained for each SNP, we calculated R^2^ by the following formula: R^2^ = 2 × MAF × (1 − MAF) × Beta^2^. Then, we summed the R^2^ to calculate the overall R^2^ and F statistics for exposure (F_statistic = R^2^ × (N − 2)/(1 − R^2^)). N means the number of individuals of the GWAS-exposure [43]. The higher the R^2^ and F statistics are, the lower the risk of weak IV bias [44]. An online mRnd tool (https://cnsgenomics.com/shiny/mRnd/) proposed by Brion et al. [45] was used to calculate the statistical power of the MR study with the input of several parameters (total sample size of the outcome, proportion of cases in the outcome study, true odds ratio of the outcome variable per standard deviation of the exposure variable, and the proportion of variance in exposure variable explained by selected SNPs).

### Heritability and genetic correlation analyses

LDSC analysis regressed χ^2^ statistics for one trait to calculate SNP-based heritability (h^2^) or two traits to estimate SNP-based coheritability (http://ldsc.broadinstitute.org/ldhub/, LD score tool, version 1.0.1). Cross-trait LDSC analysis was conducted to assess the genetic correlations between different lung function parameters and each VTE trait by the regression slope using GWAS summary data [27]. To account for multiple testing, similar to the MR analysis, we also adopted a Bonferroni correction, that is, *P* < 0.007 (0.05/7) was considered statistically significant, and 0.007 < *P* < 0.05 was considered suggestive evidence.

## Results

Detailed information on the characteristics of the SNPs used for each trait is shown in Additional file 2 (Tables S1–S7). Summary information on the GWAS data and instrumental variables is listed in Table 1.

**Table 1:** Summary information of GWAS data and instrumental variables used in our analyses

Exposures					Outcomes				
Traits	Sample size	Source	Year	Trait transformation	Trait	nIVs	R^2^	F_statistic	Power
FEV1	400,462	UKBB & SpiroMeta	2019	Raw, in liter	VTE	209	0.030	13,238.190	0.350
					DVT	209	0.030	13,238.190	0.300
					PE	213	0.031	13,373.943	0.080
FVC	400,462	UKBB & SpiroMeta	2019	Raw, in liter	VTE	189	0.029	11,468.368	0.240
					DVT	192	0.029	11,489.334	0.740
					PE	191	0.028	11,396.694	0.050
FEV1/FVC	400,462	UKBB & SpiroMeta	2019	Raw, in liter	VTE	236	0.055	22,562.624	0.130
					DVT	235	0.056	22,383.280	0.200
					PE	240	0.057	22,748.353	0.170
PEF	400,462	UKBB & SpiroMeta	2019	Raw, in liter	VTE	166	0.034	13,537.796	0.050
					DVT	168	0.034	13,679.262	0.050
					PE	168	0.034	13,679.262	0.070
VTE	9176/209,616	FinnGen Study	2021	Case control	FEV1	8	0.077	15,846.433	–
					FVC	9	0.134	30,183.011	–
					FEV1/FVC	11	0.144	32,396.142	–
					PEF	9	0.015	3312.630	–
DVT	4576/214,216	FinnGen Study	2021	Case control	FEV1	5	0.185	44,289.920	–
					FVC	5	0.185	44,289.920	–
					FEV1/FVC	6	0.195	46,644.481	–
					PEF	6	0.195	46,644.481	–
PE	4185/214,607	FinnGen Study	2021	Case control	FEV1	5	0.264	66,459.527	–
					FVC	5	0.264	66,459.527	–
					FEV1/FVC	5	0.264	66,459.527	–
					PEF	5	0.264	66,459.527	–

*FEV1* forced expiratory volume in one second; *FVC* forced vital capacity; *FEV1/FVC* the ratio of FEV1 to FVC; *PEF* peak expiratory flow; *VTE* venous thromboembolism; *DVT* deep vein thrombosis; *PE* pulmonary embolism; *nIVs* number of instrumental variables; *R*^2^ variance explained by the SNPs on exposure

### Genetic instrumental variable selection

In the forwards UVMR analyses, a total of 272/239/303/205 independent SNPs that conformed to a genome-wide significance threshold (*P* < 5 × 10^−8^) for FEV1, FVC, FEV1/FVC and PEF were filtered. Next, we extracted these SNPs from the corresponding GWAS outcomes, and there were 258/226/285/195 overlapping SNPs, respectively. Finally, 212/194/240/168 SNPs were selected as the IVs after harmonizing SNP exposures and SNP outcomes. Although the SNPs appeared to be numerous, the variance explained by these SNPs (FEV1: 0.030–0.031; FVC: 0.028–0.029; FEV1/FVC: 0.055–0.057; PEF: 0.034) and the statistical power (FEV1: 0.080–0.350; FVC: 0.050–0.740; FEV1/FVC: 0.130–0.200; PEF: 0.050–0.070) of the MR analysis were not sufficient (Table 1). The percentages of the IVs of each lung function trait associated with other lung function phenotypes are as follows: FEV1 to other phenotypes (FVC: 18.868%, FEV1/FVC: 8.491%, PEF: 9.434%), FVC to other phenotypes (FEV1: 10.309%, FEV1/FVC: 1.031%, PEF: 3.093%), FEV1/FVC to other phenotypes (FEV1: 7.500%, FVC: 0.833%, PEF: 6.250%), and PEF to other phenotypes (FEV1: 11.905%, FVC: 3.571%, FEV1/FVC: 8.929%). In the reverse UVMR analyses, we adopted the same method and finally obtained 11/5 (DVT vs. FEV1 and FVC) or 6 (DVT vs. FEV1/FVC and PEF)/6 instrumental variables. The statistical power of the reverse MR analysis was unable to be calculated because there were no previous studies that simultaneously satisfied the main parameters used for calculation. The percentages of the IVs of each venous thromboembolism subtype associated with other subtypes are as follows: VTE to DVT (50%), VTE to PE (16.667%), DVT to VTE (100%), DVT vs. PE (33.333%), PE to VTE (40%), and PE to DVT (40%). MR-Egger regression and MVMR analysis were performed to detect and correct the potential pleiotropic bias caused by overlapping IVs of different phenotypes. Please see the following sections for detailed results. The flow chart for quality control of the IVs (UVMR) is shown in Additional file 1 (Figs. S4A–D, S5A–C).

In forwards MVMR analyses, all 1019 (272 + 239 + 303 + 205) LD-independent SNPs related to FEV1, FVC, FEV1/FVC or PEF were included. Ninety-five SNPs (203 variables) represent repetitive signals, so we generated a set of SNPs (911 = 1019 − 203 + 95) by selecting the SNPs with the lowest *P* value. Next, we collected these SNPs from the corresponding GWAS outcomes, and there were 861 overlapping SNPs. Finally, 727 (727 = 861 − 134, 134 SNPs with palindromic structure) SNPs were selected as IVs after harmonizing SNP exposures and SNP outcomes. In reverse MVMR analyses, we adopted the same method, and 19 instrumental variables were obtained. The flow chart for quality control of the IVs (MVMR) is shown in Additional file 1 (Figs. S6, S7).

### Causal effect between lung function and the risk of VTE, DVT and PE via UVMR analyses

**Fig. 1:**
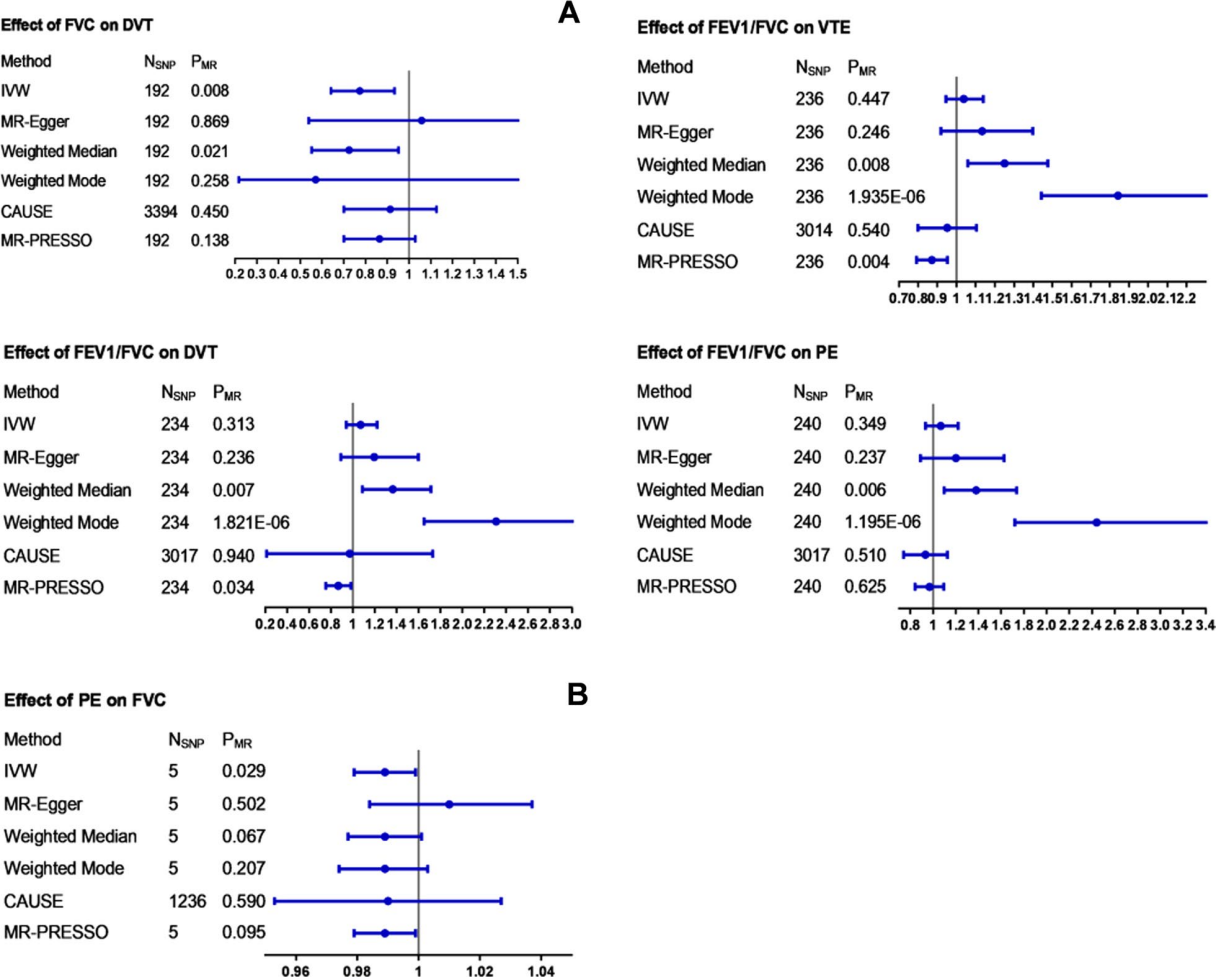
The forest plots of UVMR results. The blue dot and line represent the causal estimate and 95% confidence interval (CI), respectively. Each line represents one method to assess the potential causal effect. **A** Represents the effects of different lung function parameters on VTE risk, and **B** is the inverse direction. Causal inferences that were not nominally significant with at least two different methods are shown in Figs. S8 and S9 (Additional file 1). CAUSE filtered independent SNPs with GWAS *p* values < 1 × 10^−3^. *FEV1* forced expiratory volume in one second; *FVC* forced vital capacity; *FEV1/FVC* the ratio of FEV1 to FVC; *PEF* peak expiratory flow; *VTE* venous thromboembolism; *DVT* deep vein thrombosis; *PE* pulmonary embolism; *N*_*SNP*_ number of single nucleotide polymorphism; *IVW* inverse-variance weighted; *CAUSE* causal analysis using summary effect; *PRESSO* pleiotropy residual sum and outlier

Figures 1, S8, S9 (Additional file 1) and Tables S8, S9 (Additional file 2) show the UVMR estimates between lung function and VTE risk using different MR methods.

### Forwards UVMR analyses

#### FEV1 vs. VTE, DVT and PE

All models indicated that there was no causal effect of FEV1 on VTE, DVT and PE (VTE: OR 0.903, 95% CI 0.797–1.023, *P* = 0.110; DVT: OR 0.876, 95% CI 0.737–1.041, *P* = 0.133; PE: OR 0.953, 95% CI 0.794–1.144, *P* = 0.607) with no evidence of heterogeneity (VTE: *Q*_Pval = 0.370, *I*^2^ = 0.027; DVT: *Q*_Pval = 0.381, *I*^2^ = 0.026; PE: *Q*_Pval = 0.192, *I*^2^ = 0.034) or pleiotropy (VTE: intercept = − 0.002, *P* = 0.648; DVT: intercept = − 0.007, *P* = 0.214; PE: intercept = − 0.003, *P* = 0.679). The weighted median estimates were more accurate than the MR-Egger and weighted mode methods. CAUSE analyses indicated that the causal model was better than the sharing model of FEV1 vs. DVT (Additional file 2: Table S10), but the difference did not reach the threshold of significance (*P* = 0.340), which possibly was due to the low power of the DVT GWAS. Leave-one-out sensitivity analyses and the MR-PRESSO test did not find SNPs to alter the MR estimates, indicating the robustness and reliability of forwards UVMR results (Additional file 3: Fig. S10A–C).

#### FVC vs. VTE, DVT and PE

UVMR provided suggestive evidence for a causal effect from FVC to DVT using 192 SNPs. The OR per SD increase in FVC was 0.774 (95% CI 0.641–0.934; *P* = 0.008). In addition, the suggestive causal effect is unlikely to be affected by pleiotropy due to the limited MR-Egger intercept (− 0.006) and nonsignificant results (*P*_value = 0.346) in the reversed test. For IVs, no potential outlier SNP was detected by leave-one-out and MR-PRESSO analyses (Additional file 3: Fig. S11A–C). No evidence was found for causal associations of FVC with VTE and PE in the IVW method (VTE: OR 0.921, 95% CI 0.803–1.057, *P* = 0.241; PE: OR 0.992, 95% CI 0.812–1.211, *P* = 0.934), and neither pleiotropy (VTE: intercept = − 0.003, *P* = 0.469; PE: intercept = − 0.006, *P* = 0.416) nor heterogeneity (VTE: *Q*_Pval = 0.327, *I*^2^ = 0.041; PE: *Q*_Pval = 0.182, *I*^2^ = 0.085) was found. The weighted median estimate was suggestive of significance (OR 0.785, 95% CI 0.639–0.963, *P* = 0.020), which highlights the necessity for MVMR analysis.

#### FEV1/FVC vs. VTE, DVT and PE

According to the main IVW analyses, no causal effect of FEV1/FVC on VTE (OR 1.037, 95% CI 0.944–1.139, *P* = 0.447), DVT (OR 1.070, 95% CI 0.938–1.220, *P* = 0.313) or PE (OR 1.066, 95% CI 0.932–1.219, *P* = 0.349) was identified. The leave-one-out analysis plots are shown in Additional file 4 (Fig. S12A–C). Although MR weighted median and weighted mode estimates were significant (DVT-median: OR 1.364 (1.087–1.712), *P* = 0.007; weighted mode: OR 2.308 (1.651–3.226), *P* = 1.821e−06; PE-median: OR 1.379 (1.098–1.734), *P* = 0.006; PE-mode: OR 2.441 (1.719–3.468), *P* = 1.195e−06) or suggestive significant (VTE-median: OR 1.251, 95% CI 1.059–1.476, *P* = 0.008; VTE-mode: OR 1.843, 95% CI 1.442–2.355, *P* = 1.935e−06), overall horizontal pleiotropy effects exist (VTE: PRESSO_*P* = 0.040; DVT: intercept = − 0.003, *P* = 0.004; PE: intercept = − 0.003, *P* = 0.004), which also highlights the necessity for MVMR analysis.

#### PEF vs. VTE, DVT and PE

All models consistently indicated that genetically predicted PEF had no causal relation with VTE (OR 1.006, 95% CI 0.882–1.147, *P* = 0.928), DVT (OR 1.001, 95% CI 0.833–1.204, *P* = 0.988) or PE (OR 1.037, 95% CI 0.869–1.238, *P* = 0.687). Pleiotropy bias (VTE: intercept = 0.006, *P* = 0.159; DVT: intercept = 0.011, *P* = 0.093; PE: intercept = 0.003, *P* = 0.605) was not observed, and heterogeneity (VTE: *Q*_Pval = 0.084, *I*^2^ = 0.134; DVT: *Q*_Pval = 0.041, *I*^2^ = 0.166; PE: *Q*_Pval = 0.393, *I*^2^ = 0.025) was not detected. The main IVW estimates were generally consistent with the weighted median, weighted mode and MR-Egger sensitivity estimates. The leave-one-out sensitivity analyses (Additional file 4: Fig. S13A–C) and the MR-PRESSO analyses found that the causal relation between PEF and VTE events was not affected by any individual SNP, which indicated the reliability of the MR estimates.

### Reverse UVMR analyses

#### VTE vs. different lung function parameters

All methods in reverse UVMR analyses consistently suggested no significant causality of genetically predicted VTE with FEV1, FVC and PEF (IVW: OR_FEV1_ 1.000; 95% CI 0.989–1.012; *P* = 0.949; OR_FVC_ 0.998; 95% CI 0.998–1.008; *P* = 0.691; OR_PEF_ 1.005; 95% CI 0.994–1.016; *P* = 0.353). There was no evidence of heterogeneity between IV estimates with IVW methods from individual SNPs (FEV1: *Q*_Pval = 0.167, *I*^2^ = 0.327; FVC: *Q*_Pval = 0.153, *I*^2^ = 0.332; PE: *Q*_Pval = 0.086, *I*^2^ = 0.423) and no pleiotropy effect (FEV1: intercept = − 0.001, *P* = 0.815; FVC: intercept = − 0.003, *P* = 0.093; PE: intercept = − 0.002, *P* = 0.519). No potential outlier SNP was detected by leave-one-out and MR-PRESSO analyses (Additional file 5: Fig. S14A–C). The suggestive significant effect of VTE on FF in weighted median analysis (OR 1.012, 95% CI 1.001–1.023, *P* = 0.028) highlighted the necessity of MVMR.

#### DVT vs. different lung function parameters

According to the main IVW analyses, no causal effect of DVT on FEV1 (OR 1.005, 95% CI 0.995–1.015, *P* = 0.354), FVC (OR 1.001, 95% CI 0.993–1.008, *P* = 0.839), FEV1/FVC (OR 1.008, 95% CI 0.997–1.019, *P* = 0.144) or PEF (OR 1.007, 95% CI 0.997–1.018, *P* = 0.183) was identified. Although MR weighted median estimates were suggestive significant (FF-median: OR 1.009 (1.002–1.017), *P* = 0.012; PEF-median: OR 1.009 (1.001–1.017), *P* = 0.026), overall heterogeneity (Additional file 5: Fig. S15A–C) and horizontal pleiotropy effects exist (FF: *Q*_pval = 0.017, intercept = − 0.000, *P* = 0.004; PEF: *Q*_pval = 0.031), highlighting the necessity for MVMR analysis.

#### PE vs. different lung function parameters

According to the main IVW analyses, no causal effect of PE on FEV1 (OR 0.989, 95% CI 0.976–1.003, *P* = 0.122), FEV1/FVC (OR 0.996, 95% CI 0.985–1.008, *P* = 0.554) or PEF (OR 0.995, 95% CI 0.980–1.010, *P* = 0.482) was identified, but suggestive significance existed between PE and FVC (OR 0.989, 95% CI 0.979–0.999, *P* = 0.029). Pleiotropy bias was not detected, and heterogeneity was not found (Additional file 5: Fig. S16A–C).

None of the CAUSE analyses in reverse MR showed that the causal model was superior to the sharing model (Additional file 2: Table S11).

### Direct causal effect between lung function and the risk of VTE, DVT and PE via MVMR analyses

#### Forwards MVMR analyses

The forwards MVMR analyses indicated that the direct effect of each lung function parameter controlling for other lung function parameters on VTE was similar to the UVMR setting. Additionally, there was no direct effect of FVC (OR 0.407, 95% CI 0.018–9.067, *P* = 0.570) on DVT, which was inconsistent with the UVMR-IVW results. Consistent with the main IVW, the results of MVMR-Egger regression and MR-PRESSO (no significant outliers were detected) also showed no causal association between lung function and VTE, which suggested that the MVMR approach avoids the bias caused by horizontal pleiotropy in the UVMR analyses (Table 2).

**Table 2:** Multivariable MR for lung function on VTE (orientate to exposure FEV1)

Exposure	nSNPs	OR (95% CI)	Beta (SE)	*P*	Intercept (*P*)	*Q*_Pval (*I*^2^)	PRESSO (global *P*)
FEV1 vs. VTE					0.001 (0.477)	0.474 (0.002)	< 0.001
mvIVW	727	1.473 (0.516–4.202)	0.378 (0.535)	0.479			
mvMR-Egger	727	0.480 (0.051–4.506)					
FVC vs. VTE							
mvIVW	727	0.492 (0.052–4.615)	− 0.709 (1.142)	0.535			
mvMR-Egger	727	1.857 (0.239–14.428)					
FEV1/FVC vs. VTE							
mvIVW	727	1.885 (0.243–14.646)	0.634 (1.046)	0.544			
mvMR-Egger	727	1.449 (0.508–4.135)					
PEF vs. VTE							
mvIVW	727	0.996 (0.843–1.177)	− 0.004 (0.085)	0.965			
mvMR-Egger	727	0.984 (0.830–1.167)					
FEV1 vs. DVT					0.001 (0.499)	0.474 (0.002)	0.002
mvIVW	726	1.613 (0.377–6.892)	0.478 (0.741)	0.519			
mvMR-Egger	726	0.393 (0.018–8.747)					
FVC vs. DVT							
mvIVW	726	0.407 (0.018–9.067)	− 0.898 (1.583)	0.570			
mvMR-Egger	726	2.171 (0.126–37.299)					
FEV1/FVC vs. DVT							
mvIVW	726	2.210 (0.129–37.902)	0.793 (1.450)	0.585			
mvMR-Egger	726	1.602 (0.375–6.844)					
PEF vs. DVT							
mvIVW	726	1.006 (0.798–1.268)	0.006 (0.118)	0.959			
mvMR-Egger	726	0.990 (0.781–1.255)					
FEV1 vs. PE					0.001 (0.703)	0.484 (0.001)	0.052
mvIVW	727	1.694 (0.492–5.834)	0.527 (0.631)	0.404			
mvMR-Egger	727	0.374 (0.027–5.249)					
FVC vs. PE							
mvIVW	727	0.378 (0.027–5.302)	− 0.974 (1.348)	0.470			
mvMR-Egger	727	2.361 (0.210–26.565)					
FEV1/FVC vs. PE							
mvIVW	727	2.396 (0.213–27.020)	0.874 (1.236)	0.479			
mvMR-Egger	727	1.680 (0.488–5.788)					
PEF vs. PE							
mvIVW	727	0.991 (0.799–1.229)	− 0.009 (0.110)	0.935			
mvMR-Egger	727	0.982 (0.790–1.221)					

*FEV1* forced expiratory volume in one second; *FVC* forced vital capacity; *FEV1/FVC* the ratio of FEV1 to FVC; *PEF* peak expiratory flow; *VTE* venous thromboembolism; *DVT* deep vein thrombosis; *PE* pulmonary embolism; *MR* Mendelian randomization; *SNP* single nucleotide polymorphism; *OR* odds ratio; *CI* confidence interval; *SE* standard error; *mvIVW* multivariable inverse-variance weighted; *PRESSO* pleiotropy residual sum and outlier

#### Reverse MVMR analyses

The reverse MVMR analyses found that the direct effect of VTE or DVT controlling for another two traits on different lung functions was consistent with the UVMR analyses. Additionally, the direct effect of PE on FVC (OR 0.956, 95% CI 0.862–1.061, *P* = 0.400) was attenuated compared to the total effect obtained via UVMR. Notably, the direct causal effect of PE on FEV1 was borderline significant (OR 0.921, 95% CI 0.848–1.000, *P* = 0.050), which was inconsistent with UVMR and should be interpreted with caution (Table 3).

**Table 3:** Multivariable MR for VTE on lung function (orientate to exposure VTE)

Exposure	nSNPs	OR (95% CI)	Beta (SE)	*P*	Intercept (*P*)	*Q*_Pval (*I*^2^)	PRESSO (global *P*)
VTE vs. FEV1					− 0.004 (0.246)	0.172 (0.287)	0.375
mvIVW	13	1.039 (0.943–1.375)	0.130 (0.096)	0.173			
mvMR-Egger	13	1.120 (0.910–1.378)					
DVT vs. FEV1							
mvIVW	13	0.955 (0.869–1.049)	− 0.046 (0.048)	0.338			
mvMR-Egger	13	0.966 (0.865–1.078)					
PE vs. FEV1							
mvIVW	13	0.921 (0.848–1.000)	− 0.082 (0.042)	0.050			
mvMR-Egger	13	0.920 (0.844–1.003)					
VTE vs. FVC							
mvIVW	13	1.124 (0.915–1.381)	0.117 (0.105)	0.266			
mvMR-Egger	13	1.067 (0.860–1.324)					
DVT vs. FVC							
mvIVW	13	0.956 (0.862–1.061)	− 0.045 (0.053)	0.400	0.004 (0.197)	0.072 (0.415)	0.287
mvMR-Egger	13	0.988 (0.882–1.107)					
PE vs. FVC							
mvIVW	13	0.931 (0.850–1.018)	− 0.072 (0.046)	0.115			
mvMR-Egger	13	0.931 (0.852–1.016)					
VTE vs. FEV1/FVC					0.004 (0.063)	0.732 (0.000)	0.867
mvIVW	14	1.099 (0.937–1.288)	0.094 (0.081)	0.246			
mvMR-Egger	14	1.039 (0.876–1.232)					
DVT vs. FEV1/FVC							
mvIVW	14	0.973 (0.898–1.055)	− 0.027 (0.041)	0.516			
mvMR-Egger	14	1.007 (0.922–1.099)					
PE vs. FEV1/FVC							
mvIVW	14	0.939 (0.877–1.006)	− 0.063 (0.035)	0.074			
mvMR-Egger	14	0.943 (0.878–1.012)					
VTE vs. PEF					0.002 (0.438)	0.084 (0.375)	0.269
mvIVW	15	1.057 (0.855–1.306)	0.055 (0.108)	0.612			
mvMR-Egger	15	1.024 (0.814–1.288)					
DVT vs. PEF							
mvIVW	15	0.996 (0.894–1.109)	− 0.004 (0.055)	0.935			
mvMR-Egger	15	1.014 (0.899–1.143)					
PE vs. PEF							
mvIVW	15	0.950 (0.867–1.042)	− 0.051 (0.047)	0.277			
mvMR-Egger	15	0.953 (0.868–1.047)					

*FEV1* forced expiratory volume in one second; *FVC* forced vital capacity; *FEV1/FVC* the ratio of FEV1 to FVC; *PEF* peak expiratory flow; *VTE* venous thromboembolism; *DVT* deep vein thrombosis; *PE* pulmonary embolism; *SNP* single nucleotide polymorphism; *OR* odds ratio; *CI* confidence interval; *SE* standard error; *mvIVW* multivariable inverse-variance weighted; *PRESSO* pleiotropy residual sum and outlier

#### LDSC regression analyses

**Fig. 2:**
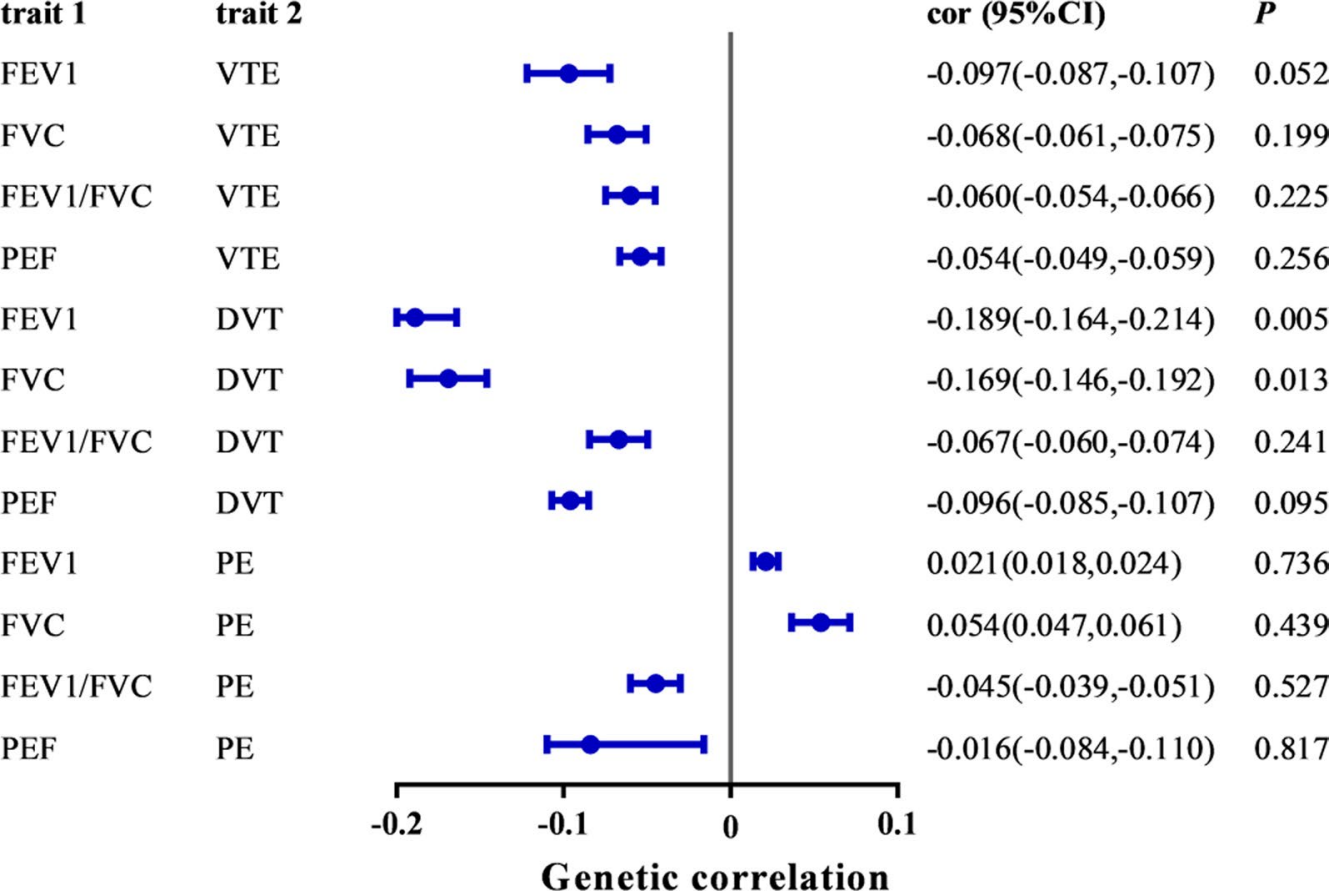
The forest plot of genetic correlation analyses. The blue dot and line represent the genetic correlation estimate and 95% confidence interval (CI), respectively. *FEV1* forced expiratory volume in one second; *FVC* forced vital capacity; *FEV1/FVC* the ratio of FEV1 to FVC; *PEF* peak expiratory flow; *VTE* venous thromboembolism; *DVT* deep vein thrombosis; *PE* pulmonary embolism; *cor* correlation coefficient

The total heritability of lung function was 11.4–15.5% (mean χ^2^ = 1.912–2.284). The total heritability of VTE (0.6–1.3%, mean χ^2^ = 1.053–1.091) was relatively small. Lung functions were not genetically correlated with VTE and PE, but significant coheritability of FEV1 with DVT (rg = − 0.189,* P* = 0.005) and suggestive coheritability of FVC with DVT (rg = − 0.169,* P* = 0.013) were found, suggesting that the causal relation between FVC and DVT may be influenced by coheritability (Fig. 2, Additional file 2: Table S12).

## Discussion

Impaired lung function is related to a higher risk of VTE events. However, whether there is a causal relation between different lung function parameters and VTE events and the direction of the causal relation are unclear. Benefitting from the large sample-based GWAS data and less-biased MR approaches, we used UVMR and MVMR to assess the total and direct effects of different lung function parameters on VTE outcomes and vice versa. Higher FVC had a beneficial total effect on DVT outcome, while a higher risk of PE had a suggestive total effect on decreased FVC outcome. Nevertheless, the direct effects for these two traits were greatly attenuated compared to the total effects (except for the borderline direct effect of PE on FEV1). Additionally, we also identified the significant coheritability of FEV1 with DVT via LDSC analysis.

VTE generally refers to DVT and PE. The lower extremities are the most common site for DVT, while PE occurs in pulmonary arteries when thrombi dislodge from the vein walls and travel with the blood into the pulmonary arteries [46]. Blood flow change, hypercoagulability and vessel wall damage are three critical factors for the pathogenesis of thrombosis [47]. Lung function, including airway flow, capacities, and oxygenation, is functionally divided into obstructive and restrictive dysfunction [48]. The physiological correlation of the heart, lung and vessels intuitively hints that any impairment of lung function may influence the health of vessels [48]. Conversely, damage to vessels, especially pulmonary vessels, may lead to pulmonary dysfunction [49]. Such conjectures were verified to some extent in our LDSC results. Several observational studies pointed out that COPD was diagnosed when lung function parameters began to decrease, was characterized by airway limitation and was related to a higher risk of VTE events [12, 50, 51]. Additionally, a prospective population-based study indicated that airway obstruction can increase the risk of VTE events (provoked or unprovoked), and decreased FEV1 and FEV1/FVC, indices of obstructive ventilation dysfunction, were related to a higher risk of VTE events, especially PE [11]. However, the heterogeneity of these observational studies, with different levels of adjustment for confounding factors (age, sex and smoking, etc.), which are known facts that could affect the development of pulmonary dysfunction and VTE events, was the critical factor impacting the reliability of the results.

Unsurprisingly, our MR analysis did not draw causal inferences consistent with observational studies, which was similar to our previously published negative results for causal inference of lung function and atrial fibrillation [52]. First, a possible reason is the heterogeneity adjusting for potential confounders or concomitant and secondary risk factors (e.g., surgery, cancer, lower extremity varices, immobilization, bronchial superinfection or right ventricular failure [51]). Furthermore, in addition to the aforementioned cancer-related and hospitalization-related VTE, unprovoked VTE also accounts for 20–30% of the disease burden of VTE [3]. Patients with unprovoked VTE are younger, which is consistent with the estimation of the higher attributable risk for genetic factors in younger patients, while the attributable risk of some specific genetic conditions in elderly patients is only approximately 7–22% [3]. Therefore, the age composition of the population may also be one of the potential reasons affecting our results. In addition, patients with COPD or pulmonary dysfunction are habitually given CT scans to assist in the diagnosis, causing referral bias, i.e., being more likely to be diagnosed with PE [11, 53], which may further affect the reliability of causal inference. Since we were unable to obtain individual-level data from multiple cohorts, this study could not complete MR analyses stratified by age, concomitant risk factors or diagnosis mode. Second, emerging evidence suggests that there is a bidirectional association between chronic inflammation and thrombosis [10, 54]. Meanwhile, patients with different degrees of impaired lung function exhibited different degrees of systemic inflammation, and the inflammatory response was proportional to the deterioration of lung function and disease progression [55]. The potential mechanisms of coagulopathy related to chronic inflammation have been proposed but are not well defined [10], and pulmonary hypertension with venous stasis secondary to impaired lung function and hypoxia are potential risk mediators [11]. In addition, a prospective cohort study indicated that patients with respiratory symptoms but normal lung functions also had a higher risk of VTE events (especially provoked VTE) [11]. Therefore, lung function may represent only manifestations or markers for some comorbidities rather than decisive causal risk factors for VTE. Third, genetic correlation between lung function and VTE events may be another potential reason because pleiotropic effects may exist in addition to causality. An observational study showed that FEV1 is a feature of the severity of obstruction ventilation dysfunction that can identify undiagnosed COPD, while FVC represents overall vital capacity [56], higher FEV1/FVC is a feature of restriction, lower FEV1/FVC represents airflow obstruction [57], and reduced PEF is a physiological change in older individuals [58]. Genetically, the multiple gene regions related to FEV1 and FEV1/FVC play roles in biological pathways of inflammation [22, 59]. Thus, the same genetic mechanisms may determine or influence different lung function parameters, which may violate the assumption and bias the estimates from UVMR analysis. Our present study successfully accounted for the aforementioned bias, which was consistent with the LDSC results to some extent. Although the causal association was not significant and the underlying mechanism between lung function and VTE events remains unclear, our study provided suggestive genetic evidence for a clinical concern to support the importance of lung function (especially FVC) assessments in monitoring and preventing the risk of VTE events (especially PE), and lung function parameters may be a useful marker of a higher risk of VTE events. In addition, individuals with low lung capacity (FEV1 or FVC) should pay special attention to and reduce unhealthy lifestyles related to DVT, such as sedentary long the station or long time to maintain a posture. Finally, although a large sample cohort was used, the low statistical power of this MR analysis may be a potential reason for failure to detect a true causal relationship. Therefore, the summary data of a larger sample size need to be discovered, and the MR results should be evaluated in an independent population to verify their reliability, if possible.

The design of the MR study is less susceptible to potential confounders and inverse causality, but limitations exist. First, the statistical powers of the reverse MR analyses were unable to be calculated because the main parameters could not be obtained. Therefore, the MR results should be interpreted cautiously with reference to the existing evidence of different study designs. Second, the summary GWAS data used in this study were derived from a European population, so our conclusions may not generalize to other ethnic populations. Third, although lung function and VTE GWASs have no cohort overlap, they might have some sample overlap, which would inflate the Type 1 error rate [31]. Furthermore, the two GWASs we used were both adjusted for age and sex, which could produce collider bias and lead to the identification of invalid IVs affecting the interpretability and validity of the results [60]. Fourth, limited by current knowledge and the inability to obtain summary data for GWAS of VTE risk factors to assess potential genetic correlations, we cannot avoid the possibility of pleiotropy effects. Nonetheless, we performed MR-Egger regression and CAUSE analyses, which were more robust to invalid SNPs and considered the correlated and uncorrelated pleiotropy effects. Fifth, the identified SNPs may exhibit potential weak instrument bias, but this is less likely because the F statistics for each SNP used were significantly higher than ten.

## Conclusions

Our findings identified the coheritability of FEV1 (significant) and FVC (suggestive) with DVT. There was no convincing causality between lung function and the risk of VTE or its subtypes. Nevertheless, the borderline causal effect of PE on FEV1 and the significant genetic correlation between FEV1 and DVT may have clinical implications for improving the quality of existing prevention and intervention strategies. The identification of patients at higher risk for VTE events may lead to a more targeted preventive treatment of those individuals. Additionally, MR studies using individual-level statistics may be beneficial to elucidate the potential nonlinear relation between lung function and VTE events.

## Supplementary Information

**Additional file 1: Figure S1.** The possible explanations for the association of SNPs with FEV1 exposure and VTE outcomes. **Figure S2.** Schematic diagram of MVMR analysis. **Figure S3.** Schematic diagram of the study design. **Figure S4. A** Flow chart for quality control of the instrumental variables (FEV1) for forwards UVMR analyses. **B** Flow chart for quality control of the instrumental variables (FVC) for forwards UVMR analyses. **C** Flow chart for quality control of the instrumental variables (FEV1/FVC) for forwards UVMR analyses. **D** Flow chart for quality control of the instrumental variables (PEF) for forwards UVMR analyses. **Figure S5. A** Flow chart for quality control of the instrumental variables (VTE) for reverse UVMR analyses. **B** Flow chart for quality control of the instrumental variables (DVT) for reverse UVMR analyses. **C** Flow chart for quality control of the instrumental variables (PE) for reverse UVMR analyses. **Figure S6.** Flow chart for quality control of the instrumental variables for forwards MVMR analyses. **Figure S7.** Flow chart for quality control of the instrumental variables for reverse MVMR analyses. **Figure S8.** Forest plots of forwards UVMR. **Figure S9.** Forest plots of reverse UVMR.

**Additional file 2: Table S1.** SNPs for FEV1 in the forwards MR analyses: Harmonized Data (r^2^ < 0.001). **Table S2.** SNPs for FVC in the forwards MR analyses: Harmonized Data (r^2^ < 0.001). **Table S3.** SNPs for FEV1/FVC in the forwards MR analyses: Harmonized Data (r^2^ < 0.001). **Table S4.** SNPs for PEF in the forwards MR analyses: Harmonized Data (r^2^ < 0.001). **Table S5.** SNPs for VTE in the reverse MR analyses: Harmonized Data (r^2^ < 0.001). **Table S6.** SNPs for DVT in the reverse MR analyses: Harmonized Data (r^2^ < 0.001). **Table S7.** SNPs for PE in the reverse MR analyses: Harmonized Data (r^2^ < 0.001). **Table S8.** Causal associations of lung function with the risk of VTE by forwards UVMR analyses. **Table S9.** Causal associations of VTE with lung function by reverse UVMR analyses. **Table S10.** The results of CAUSE analyses via forwards UVMR. **Table S11.** The results of CAUSE analyses via reverse UVMR. **Table S12.** Heritability and genetic correlations of lung function and VTE.

**Additional file 3: Figure S10. A** MR leave-one-out sensitivity analysis for FEV1 on VTE. **B** MR leave-one-out sensitivity analysis for FEV1 on DVT. **C** MR leave-one-out sensitivity analysis for FEV1 on PE. **Figure S11. A** MR leave-one-out sensitivity analysis for FVC on VTE. **B** MR leave-one-out sensitivity analysis for FVC on DVT. **C** MR leave-one-out sensitivity analysis for FVC on PE.

**Additional file 4: Figure S12. A** MR leave-one-out sensitivity analysis for FEV1/FVC on VTE. **B** MR leave-one-out sensitivity analysis for FEV1/FVC on DVT. **C** MR leave-one-out sensitivity analysis for FEV1/FVC on PE. **Figure S13. A** MR leave-one-out sensitivity analysis for PEF on VTE. **B** MR leave-one-out sensitivity analysis for PEF on DVT. **C** MR leave-one-out sensitivity analysis for PEF on PE.

**Additional file 5: Figure S14. A** MR leave-one-out sensitivity analysis for VTE on FEV1. **B** MR leave-one-out sensitivity analysis for VTE on FVC. **C** MR leave-one-out sensitivity analysis for VTE on FEV1/FVC. **D** MR leave-one-out sensitivity analysis for VTE on PEF. **Figure S15. A** MR leave-one-out sensitivity analysis for DVT on FEV1. **B** MR leave-one-out sensitivity analysis for DVT on FVC. **C** MR leave-one-out sensitivity analysis for DVT on FEV1/FVC. **D** MR leave-one-out sensitivity analysis for DVT on PEF. **Figure S16. A** MR leave-one-out sensitivity analysis for PE on FEV1. **B** MR leave-one-out sensitivity analysis for PE on FVC. **C** MR leave-one-out sensitivity analysis for PE on FEV1/FVC. **D** MR leave-one-out sensitivity analysis for PE on PEF.

## Data Availability

All GWAS summary statistics in the current study are publicly available, and all corresponding links are included in this manuscript and its references.

## Abbreviations

CAUSE: Causal analysis using summary effect estimates
CI: Confidence interval
COPD: Chronic obstructive pulmonary disease
DVT: Deep vein thrombosis
FEV1: Forced expiratory volume in one second
FEV1/FVC: The ratio of FEV1 to FVC
FVC: Force vital capacity
GWAS: Genome-wide association studies
IVs: Instrumental variables
IVW: Inverse variance-weighted
LDSC: Linkage disequilibrium score regression
MR: Mendelian randomization
MR-PRESSO: MR pleiotropy residual sum and outlier
MVMR: Multivariate MR
OR: Odds ratio
PE: Pulmonary embolism
PEF: Peak expiratory flow
RCT: Randomized controlled trial
SD: Standard deviation
SE: Standard error
SNPs: Single nucleotide polymorphisms
UVMR: Univariate MR
VTE: Venous thromboembolism
