# Supplementary material for: Genetic association and causal inference between lung function and venous thromboembolism
Source: Respir Res. 2023 Jan 30;24:36. doi: 10.1186/s12931-023-02335-3 (PMC9885683; doi:10.1186/s12931-023-02335-3)
Supplement: Supplementary file 1 — Additional file 1: Figure S1. The possible explanations for the association of SNPs with FEV1 exposure and VTE outcomes. Figure S2. Schematic diagram of MVMR analysis. Figure S3. Schematic diagram of the study design. Figure S4. A Flow chart for quality control of the instrumental variables (FEV1) for forwards UVMR analyses. B Flow chart for quality control of the instrumental variables (FVC) for forwards UVMR analyses. C Flow chart for quality control of the instrumental variables (FEV1/FVC) for forwards UVMR analyses. D Flow chart for quality control of the instrumental variables (PEF) for forwards UVMR analyses. Figure S5. A Flow chart for quality control of the instrumental variables (VTE) for reverse UVMR analyses. B Flow chart for quality control of the instrumental variables (DVT) for reverse UVMR analyses. C Flow chart for quality control of the instrumental variables (PE) for reverse UVMR analyses. Figure S6. Flow chart for quality control of the instrumental variables for forwards MVMR analyses. Figure S7. Flow chart for quality control of the instrumental variables for reverse MVMR analyses. Figure S8. Forest plots of forwards UVMR. Figure S9. Forest plots of reverse UVMR. [file 12931_2023_2335_MOESM1_ESM.docx]

**Additional file 1**


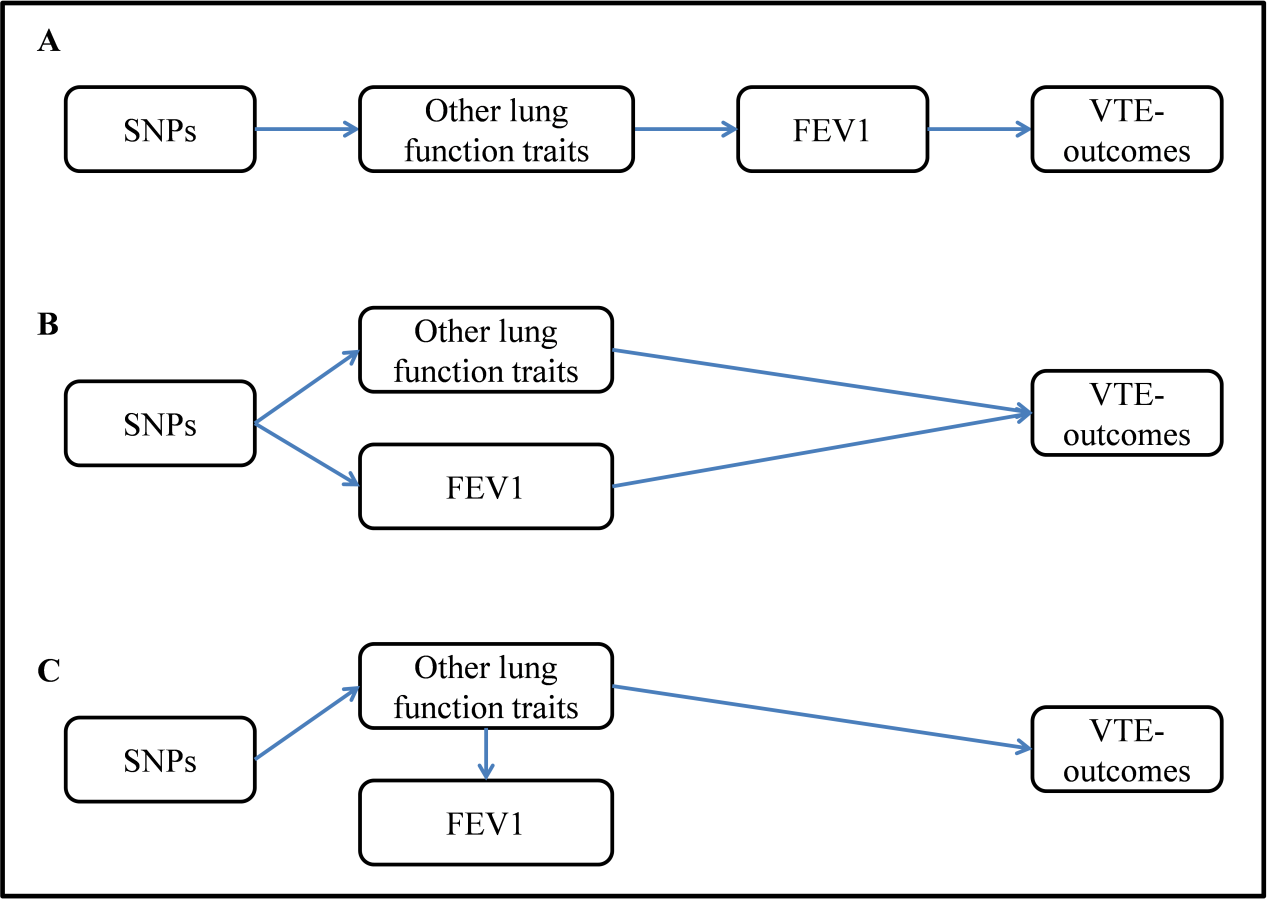


**Figure S1** The possible explanations for the association of SNPs with FEV1 exposure and VTE outcomes.

A: vertical pleiotropy: SNPs robustly related to FEV1 affect VTE outcomes via other lung function traits firstly, and then downstream affect FEV1. B: horizontal pleiotropy: SNPs related to FEV1 affect VTE outcomes via other lung function traits without mediation by FEV1. C: confounding pleiotropy: SNPs affect VTE outcomes via lung function traits other than FEV1, even though SNPs affect FEV1 via other lung function parameters. SNP: single-nucleotide polymorphism; FEV1: forced expiratory volume in one second; VTE: venous thromboembolism.


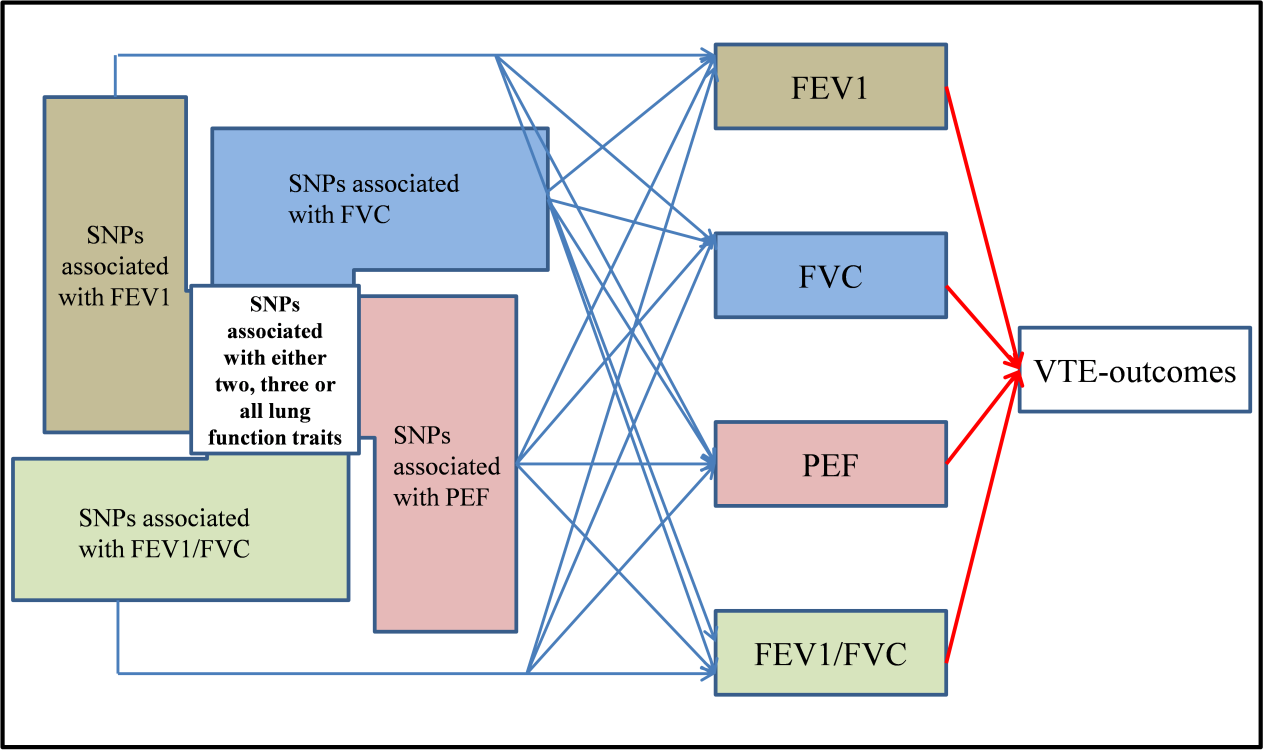


**Figure S2** Schematic diagram of MVMR analysis.

MVMR analysis estimates the direct effect (the red arrows) of different lung function traits on VTE outcomes using a list of SNPs that related to FEV1 and/or FVC and/or FEV1/FVC and/or PEF at *P* < 5×10^-8^.

FEV1: forced expiratory volume in one second; FVC: forced vital capacity; FEV1/FVC: the ratio of FEV1 to FVC; PEF: peak expiratory flow; VTE: venous thromboembolism; SNP: single-nucleotide polymorphism.


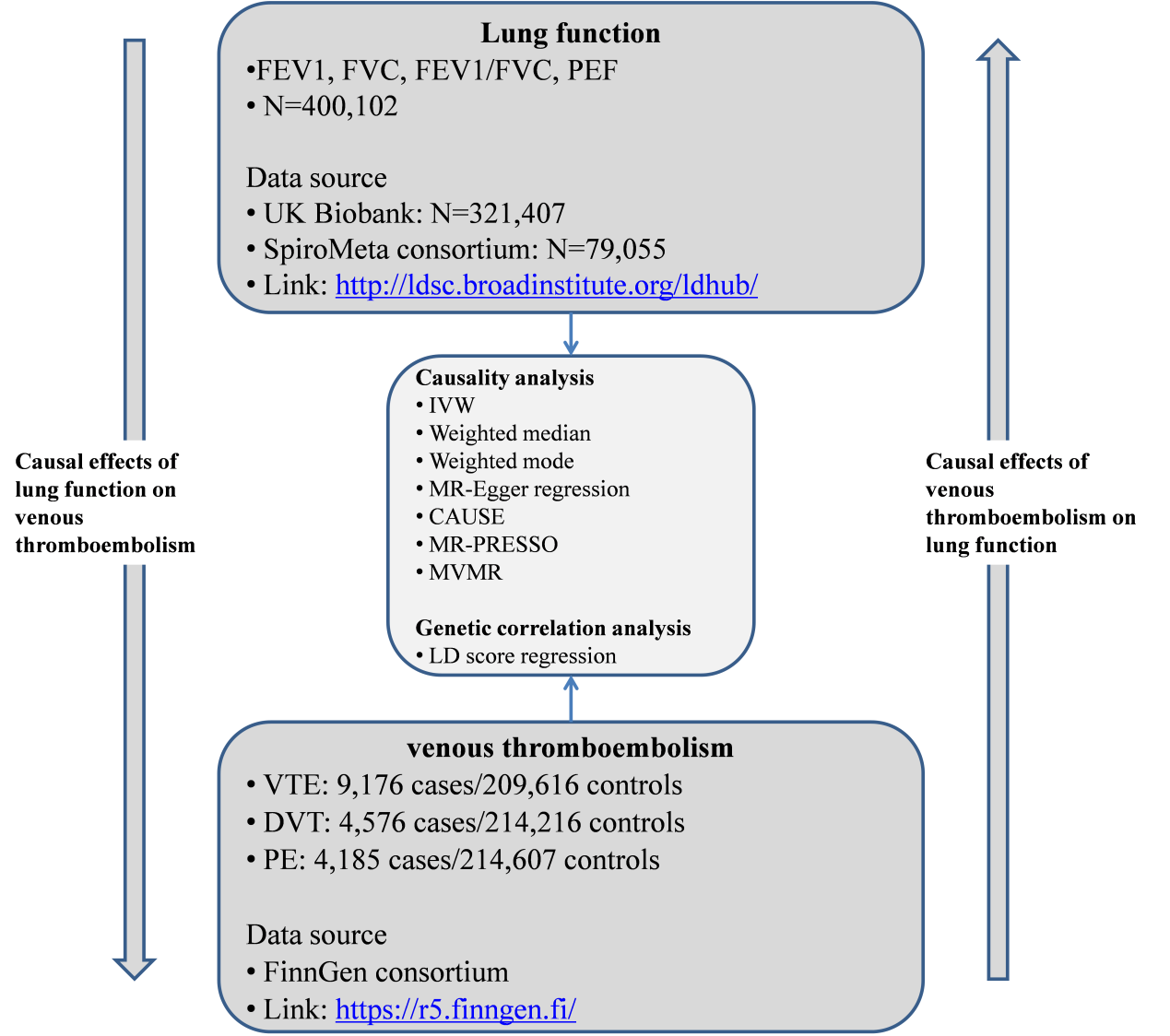


**Figure S3** Schematic diagram of the study design.

FEV1: forced expiratory volume in one second; FVC: forced vital capacity; FEV1/FVC: the ratio of FEV1 to FVC; PEF: peak expiratory flow; VTE: venous thromboembolism; DVT: deep vein thrombosis; PE: pulmonary embolism; IVW: inverse-variance weighted; MR-PRESSO: Mendelian randomization pleiotropy residual sum and outlier; CAUSE: causal analysis using summary effect.

**
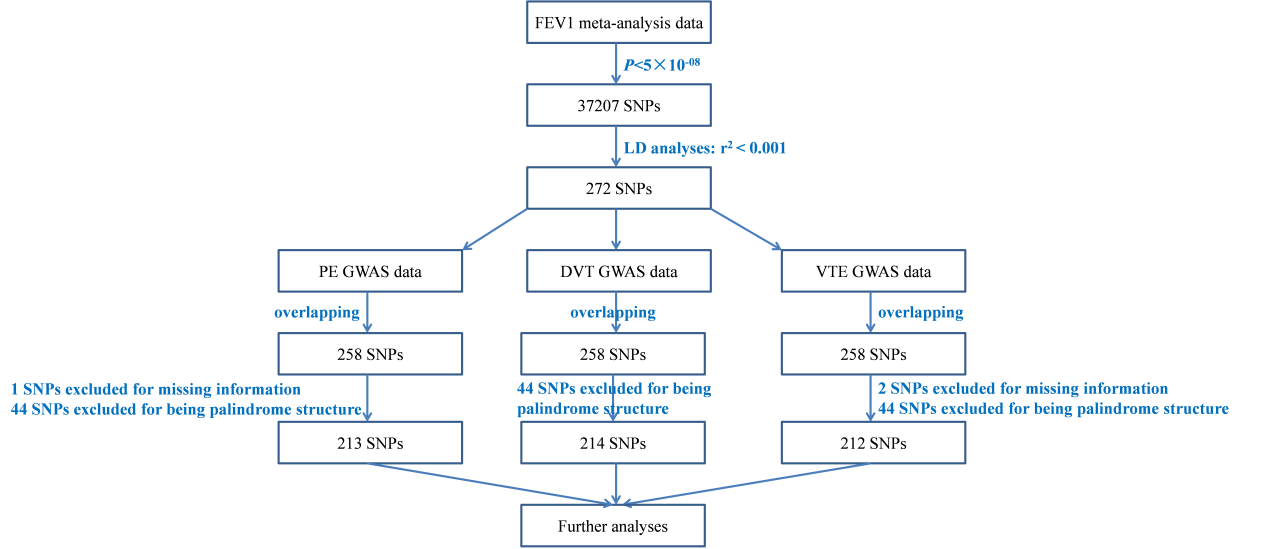
**

**Figure S4.A** Flow chart for quality control of the instrumental variables (FEV1) for forwards UVMR analyses.

FEV1: forced expiratory volume in one second; VTE: venous thromboembolism; DVT: deep vein thrombosis; PE: pulmonary embolism; GWAS: genome wide association study; SNP: single-nucleotide polymorphism; LD: linkage disequilibrium.

**
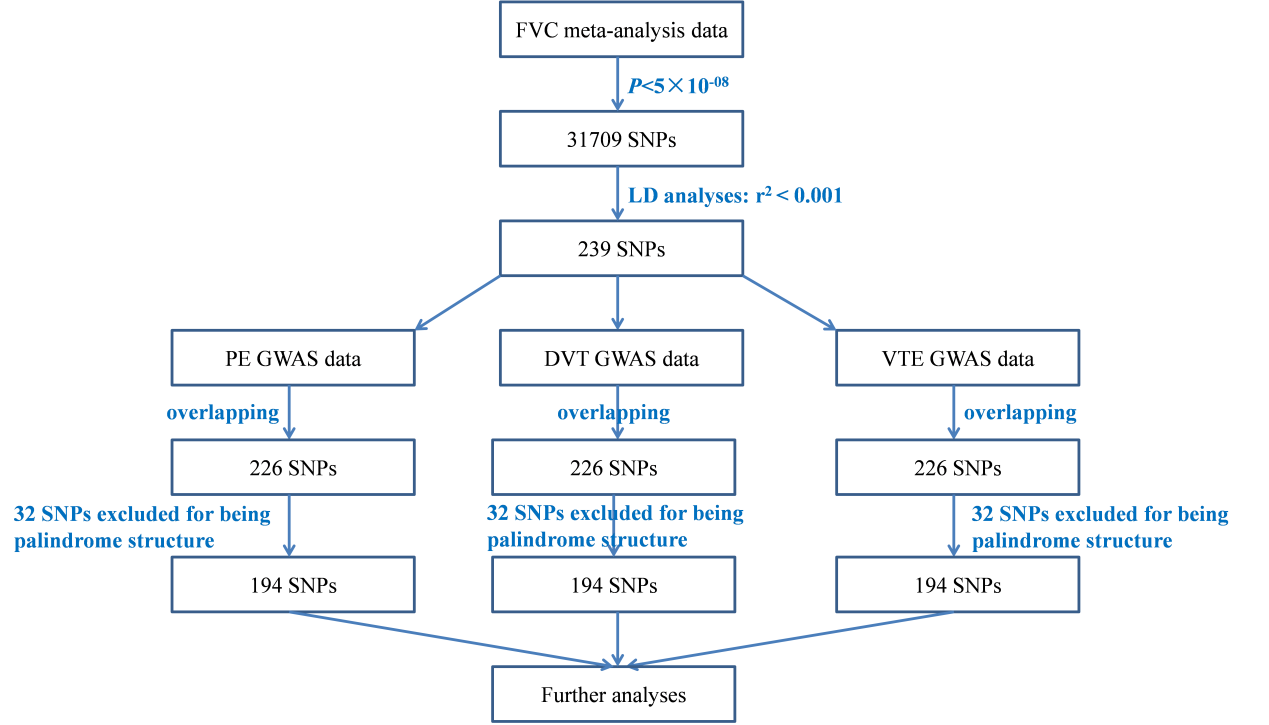
**

**Figure S4.B** Flow chart for quality control of the instrumental variables (FVC) for forwards UVMR analyses.

FVC: forced vital capacity; VTE: venous thromboembolism; DVT: deep vein thrombosis; PE: pulmonary embolism; GWAS: genome wide association study; SNP: single-nucleotide polymorphism; LD: linkage disequilibrium.


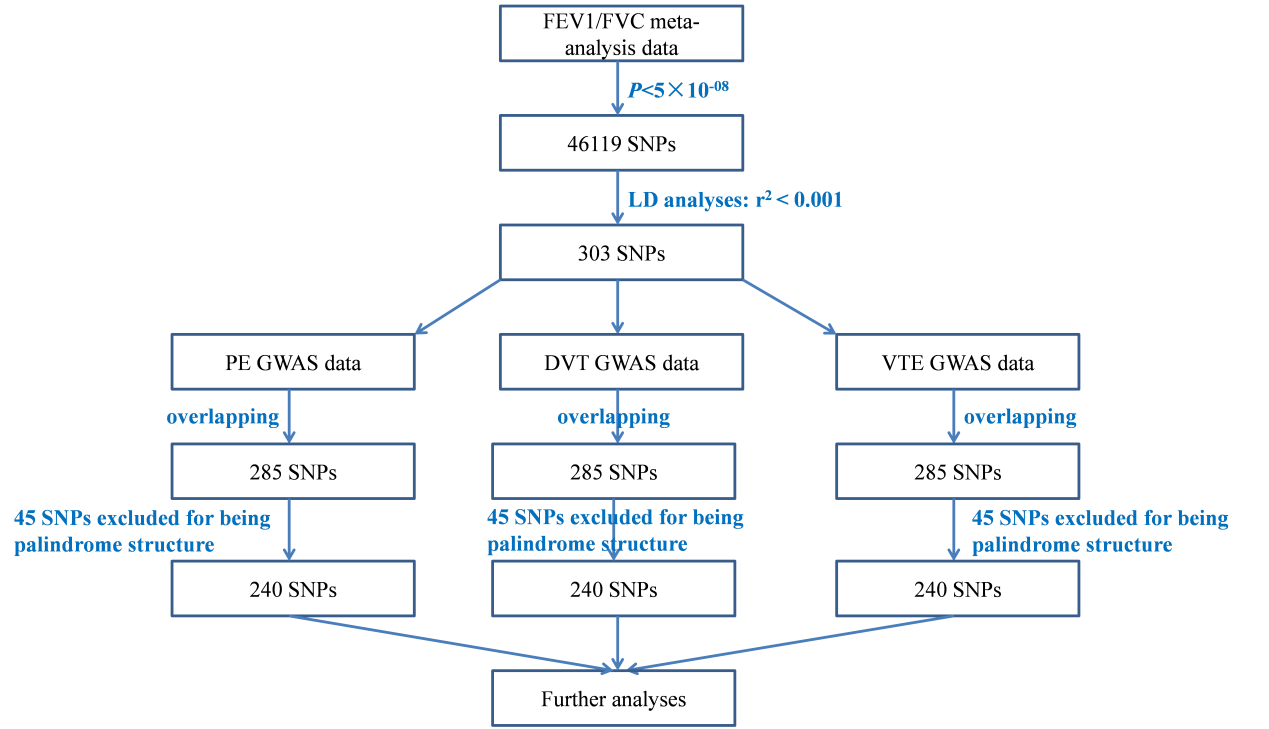


**Figure S4.C** Flow chart for quality control of the instrumental variables (FEV1/FVC) for forwards UVMR analyses.

FEV1/FVC: the ratio of FEV1 to FVC; VTE: venous thromboembolism; DVT: deep vein thrombosis; PE: pulmonary embolism; GWAS: genome wide association study; SNP: single-nucleotide polymorphism; LD: linkage disequilibrium.


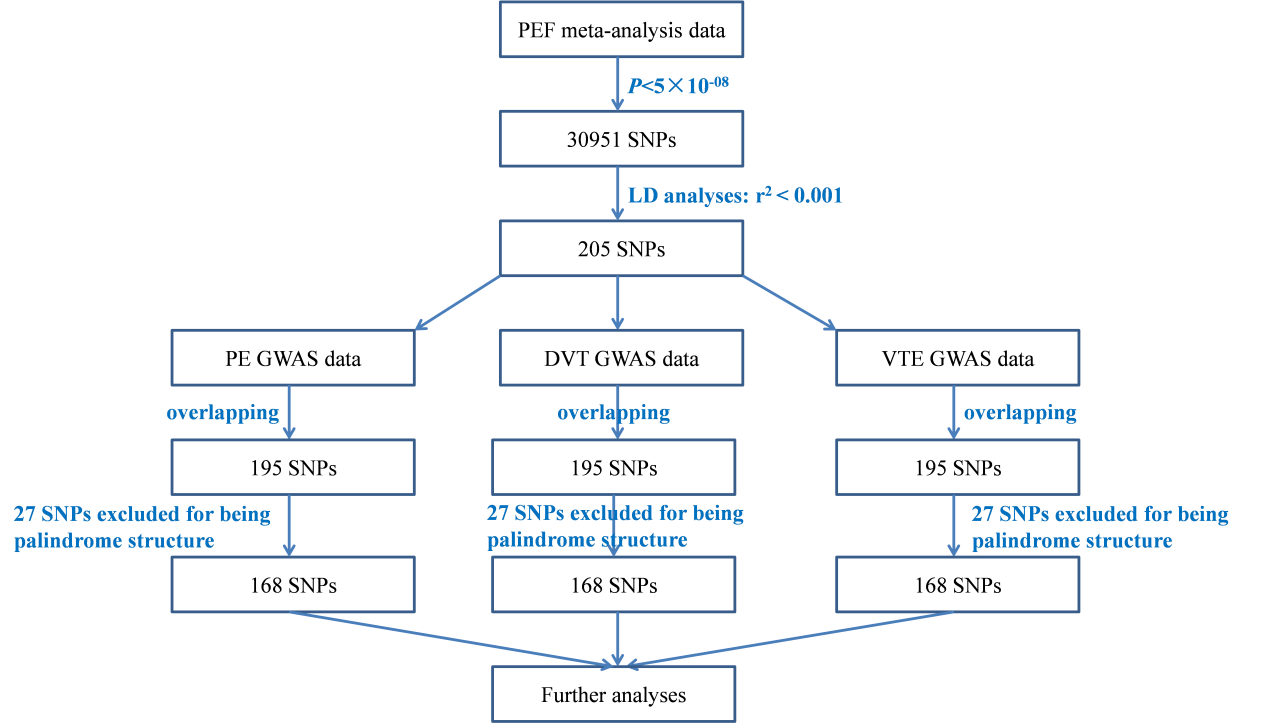


**Figure S4.D** Flow chart for quality control of the instrumental variables (PEF) for forwards UVMR analyses.

PEF: peak expiratory flow; VTE: venous thromboembolism; DVT: deep vein thrombosis; PE: pulmonary embolism; GWAS: genome wide association study; SNP: single-nucleotide polymorphism; LD: linkage disequilibrium.


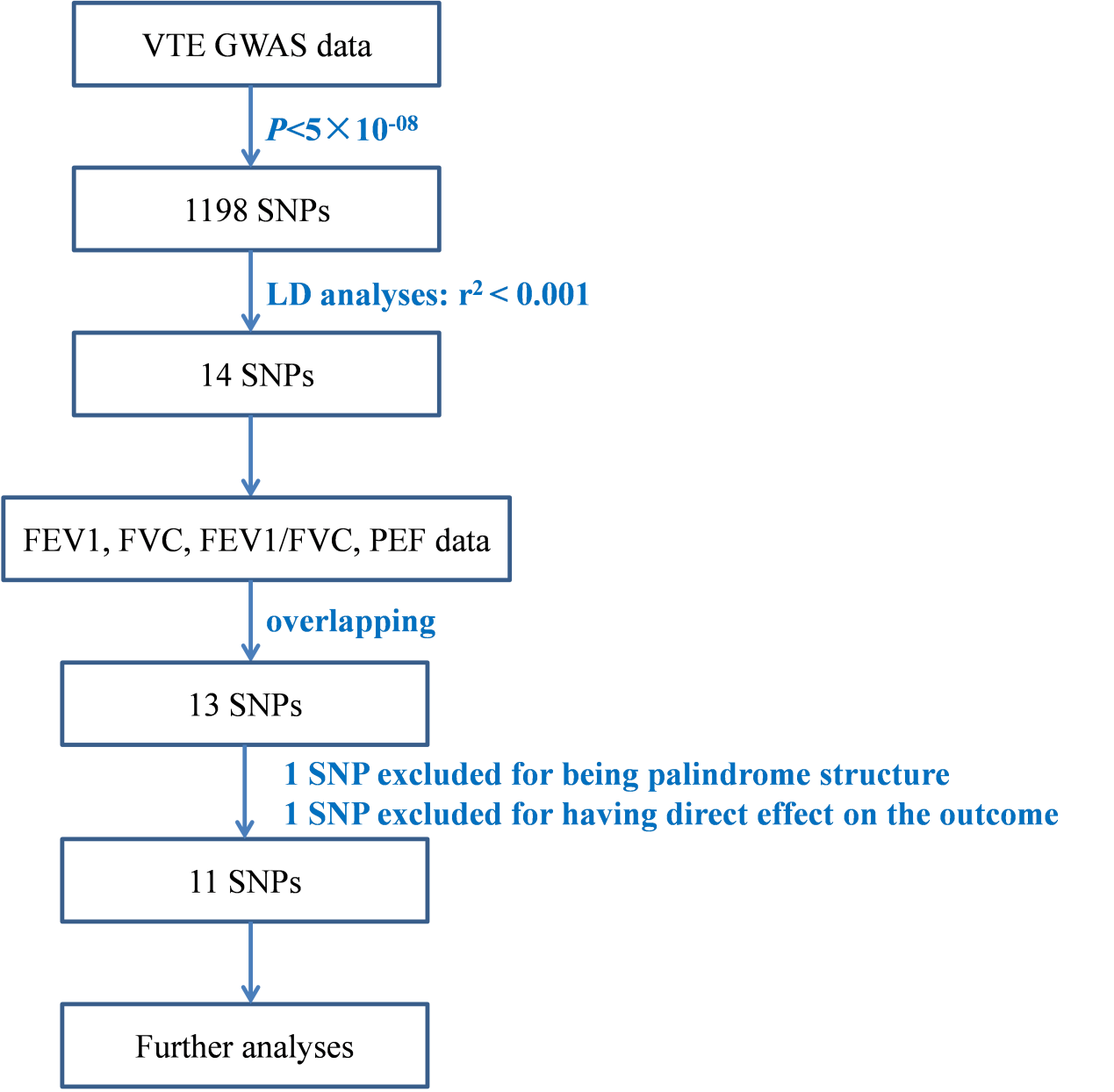


**Figure S5.A** Flow chart for quality control of the instrumental variables (VTE) for reverse UVMR analyses.

FEV1: forced expiratory volume in one second; FVC: forced vital capacity; FEV1/FVC: the ratio of FEV1 to FVC; PEF: peak expiratory flow; VTE: venous thromboembolism; GWAS: genome wide association study; SNP: single-nucleotide polymorphism; LD: linkage disequilibrium.


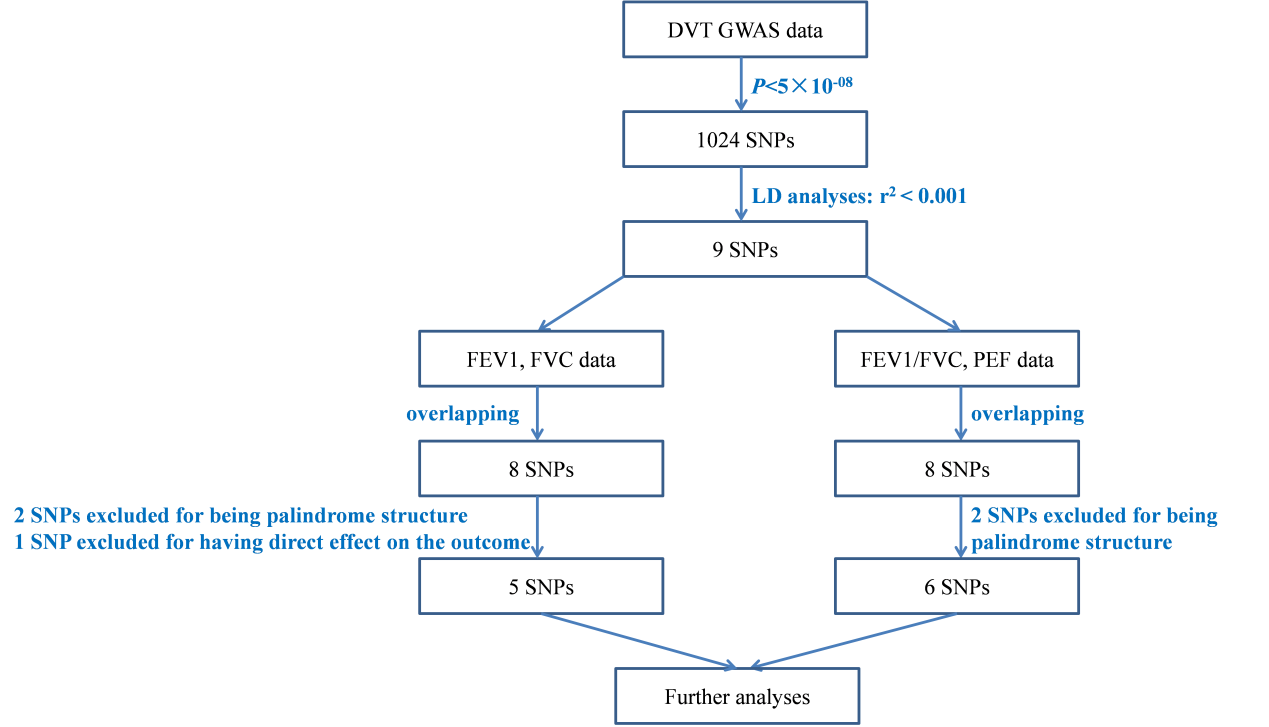


**Figure S5.B** Flow chart for quality control of the instrumental variables (DVT) for reverse UVMR analyses.

FEV1: forced expiratory volume in one second; FVC: forced vital capacity; FEV1/FVC: the ratio of FEV1 to FVC; PEF: peak expiratory flow; DVT: deep vein thrombosis; GWAS: genome wide association study; SNP: single-nucleotide polymorphism; LD: linkage disequilibrium.


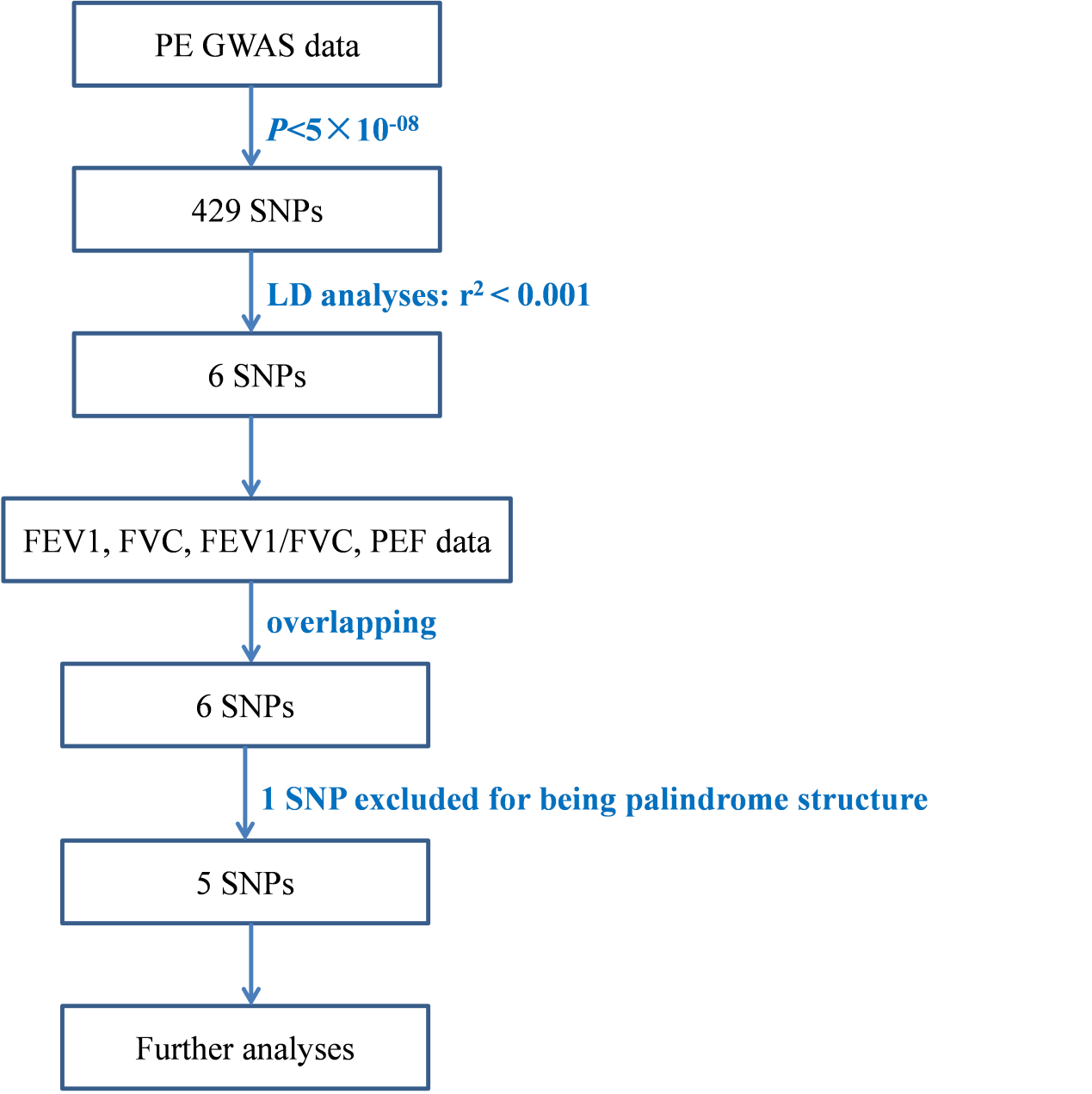


**Figure S5.C** Flow chart for quality control of the instrumental variables (PE) for reverse UVMR analyses.

FEV1: forced expiratory volume in one second; FVC: forced vital capacity; FEV1/FVC: the ratio of FEV1 to FVC; PEF: peak expiratory flow; PE: pulmonary embolism; GWAS: genome wide association study; SNP: single-nucleotide polymorphism; LD: linkage disequilibrium.


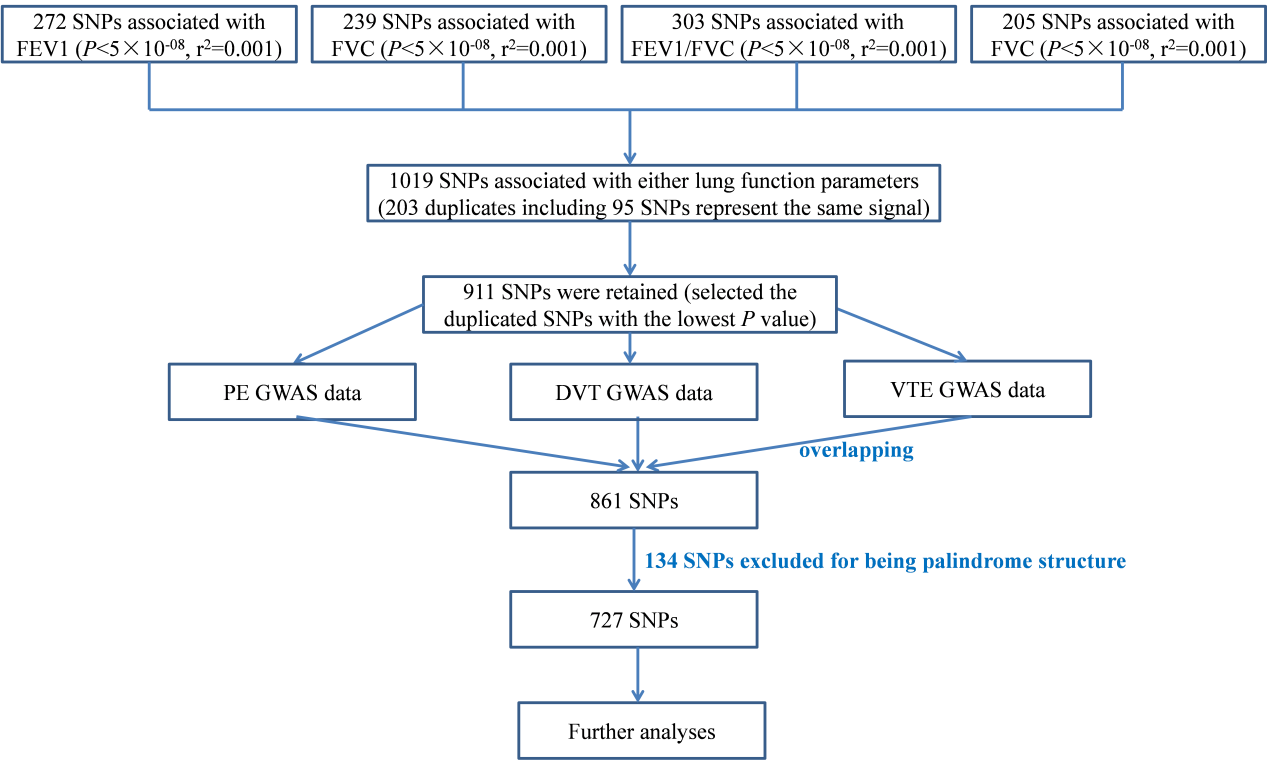


**Figure S6** Flow chart for quality control of the instrumental variables for forwards MVMR analyses.

FEV1: forced expiratory volume in one second; FVC: forced vital capacity; FEV1/FVC: the ratio of FEV1 to FVC; PEF: peak expiratory flow; VTE: venous thromboembolism; DVT: deep vein thrombosis; PE: pulmonary embolism; GWAS: genome wide association study; SNP: single-nucleotide polymorphism; LD: linkage disequilibrium.


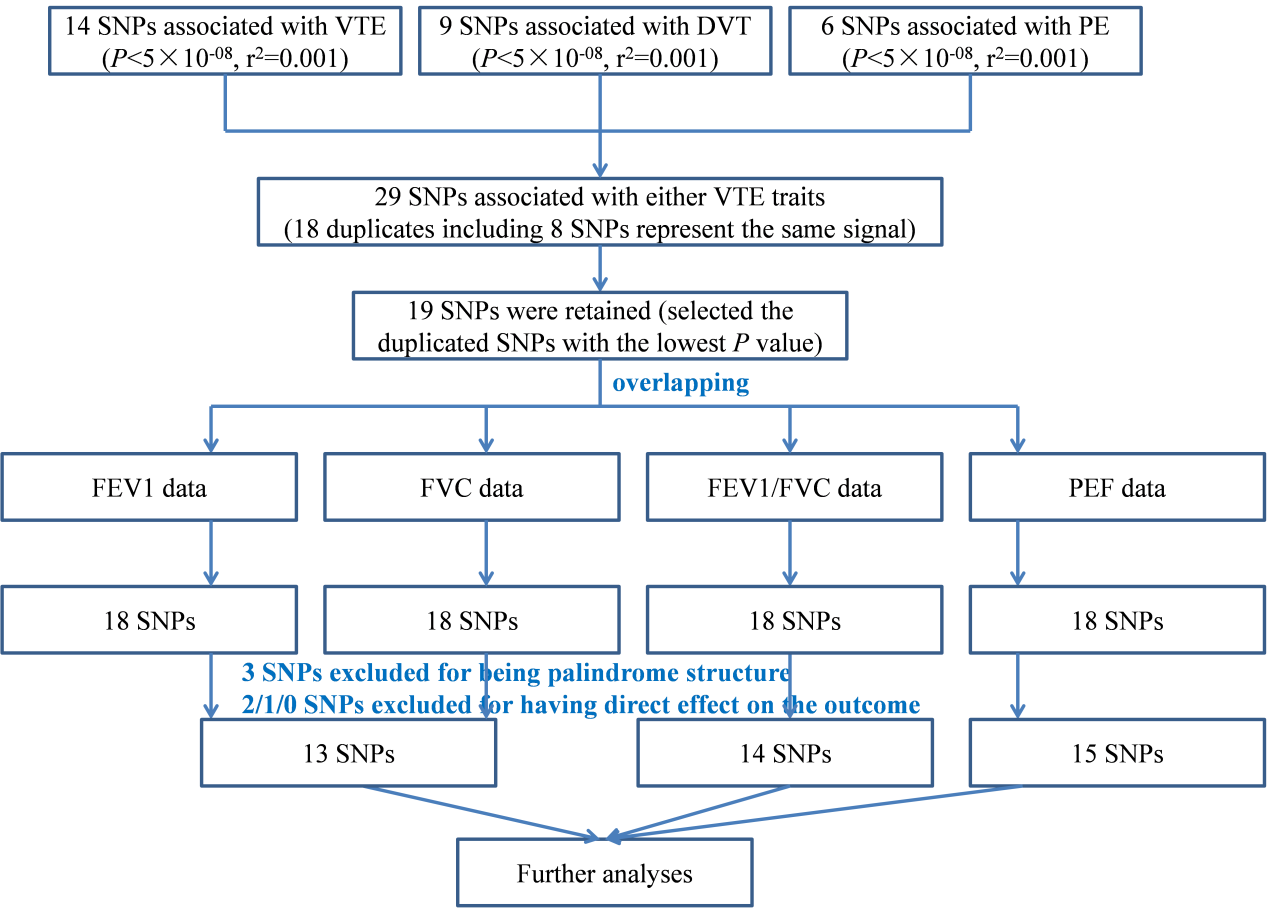


**Figure S7** Flow chart for quality control of the instrumental variables for reverse MVMR analyses.

FEV1: forced expiratory volume in one second; FVC: forced vital capacity; FEV1/FVC: the ratio of FEV1 to FVC; PEF: peak expiratory flow; VTE: venous thromboembolism; DVT: deep vein thrombosis; PE: pulmonary embolism; GWAS: genome wide association study; SNP: single-nucleotide polymorphism; LD: linkage disequilibrium.


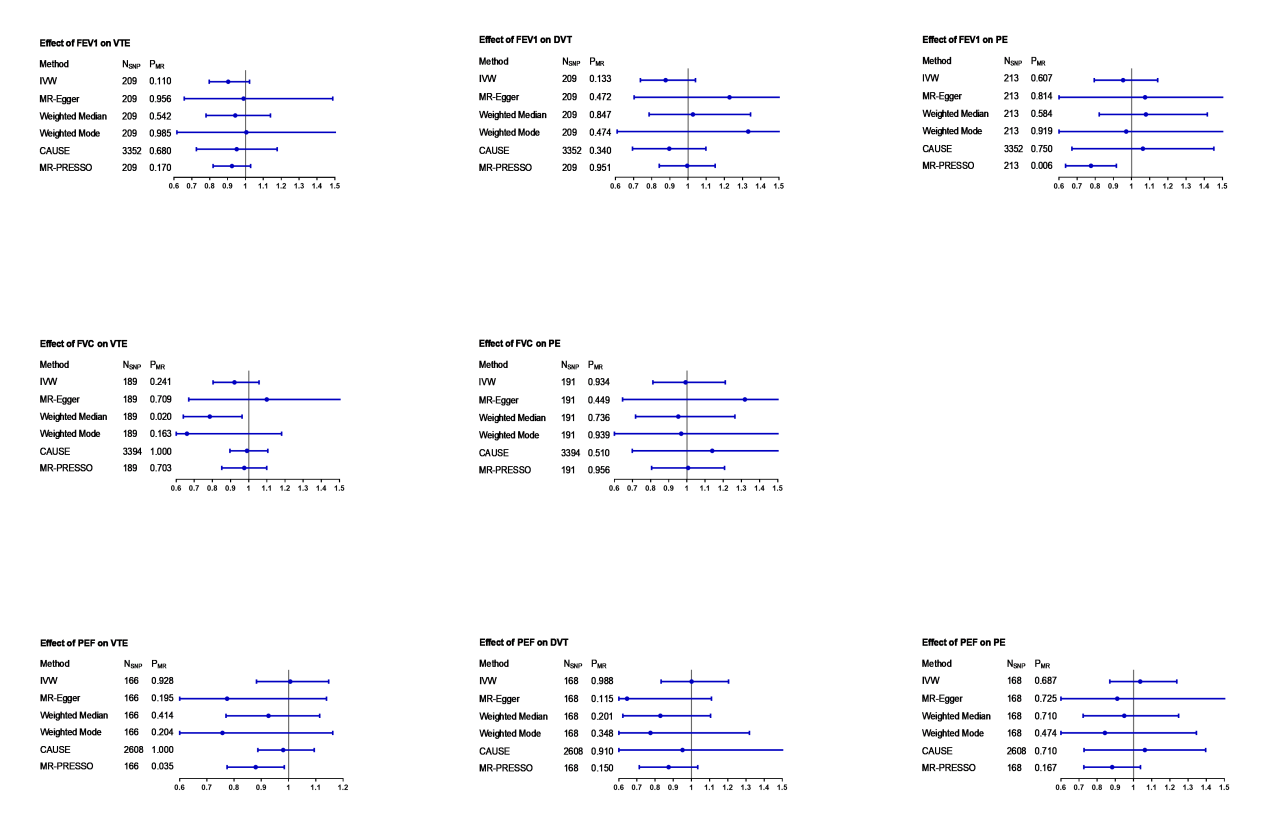


**Figure S8** Forest plots of forwards UVMR.

The blue dot and line represent the causal estimate and 95% confidence interval, respectively. Each line represents one approach to estimate the potential causal effect.

CAUSE recruited independent instrumental SNPs with GWAS *p* values < 1×10^-3^.

FEV1: forced expiratory volume in one second; FVC: forced vital capacity; FEV1/FVC: the ratio of FEV1 to FVC; PEF: peak expiratory flow; VTE: venous thromboembolism; DVT: deep vein thrombosis; PE: pulmonary embolism; N_SNP_: number of [single](javascript:;) [nucleotide](javascript:;) [polymorphism](javascript:;); IVW: inverse-variance weighted; CAUSE: causal analysis using summary effect; PRESSO: pleiotropy residual sum and outlier.


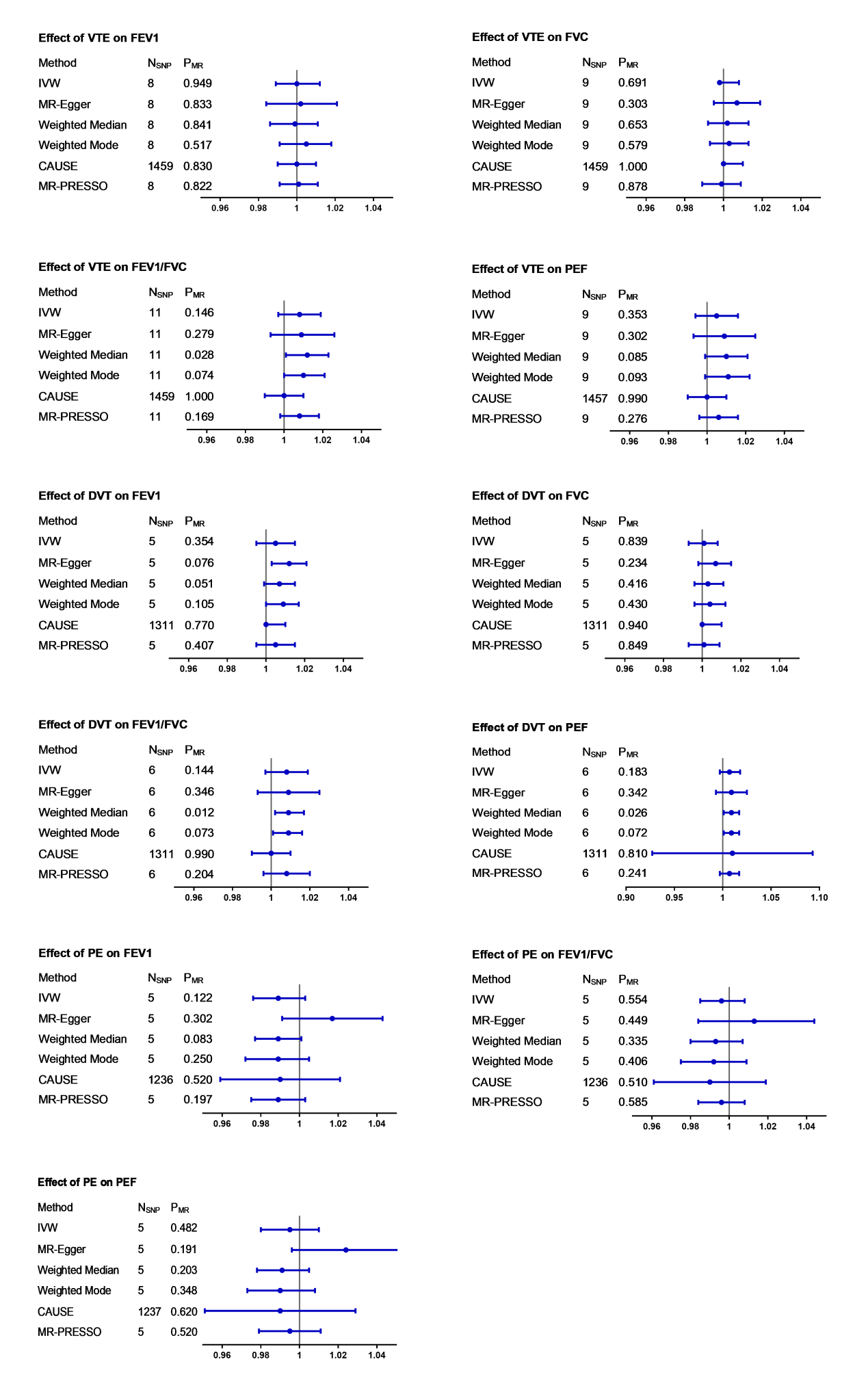


**Figure S9** Forest plots of reverse UVMR.

The blue dot and line represent the causal estimate and 95% confidence interval, respectively. Each line represents one approach to estimate the potential causal effect.

CAUSE recruited independent instrumental SNPs with GWAS *p* values < 1×10^-3^.

FEV1: forced expiratory volume in one second; FVC: forced vital capacity; FEV1/FVC: the ratio of FEV1 to FVC; PEF: peak expiratory flow; VTE: venous thromboembolism; DVT: deep vein thrombosis; PE: pulmonary embolism; N_SNP_: number of [single](javascript:;) [nucleotide](javascript:;) [polymorphism](javascript:;); IVW: inverse-variance weighted; CAUSE: causal analysis using summary effect; PRESSO: pleiotropy residual sum and outlier.
