# Supplementary material for: Genetic association and causal inference between lung function and venous thromboembolism
Source: Respir Res. 2023 Jan 30;24:36. doi: 10.1186/s12931-023-02335-3 (PMC9885683; doi:10.1186/s12931-023-02335-3)
Supplement: Supplementary file 3 — Additional file 3: Figure S10. A MR leave-one-out sensitivity analysis for FEV1 on VTE. B MR leave-one-out sensitivity analysis for FEV1 on DVT. C MR leave-one-out sensitivity analysis for FEV1 on PE. Figure S11. A MR leave-one-out sensitivity analysis for FVC on VTE. B MR leave-one-out sensitivity analysis for FVC on DVT. C MR leave-one-out sensitivity analysis for FVC on PE. [file 12931_2023_2335_MOESM3_ESM.pdf]

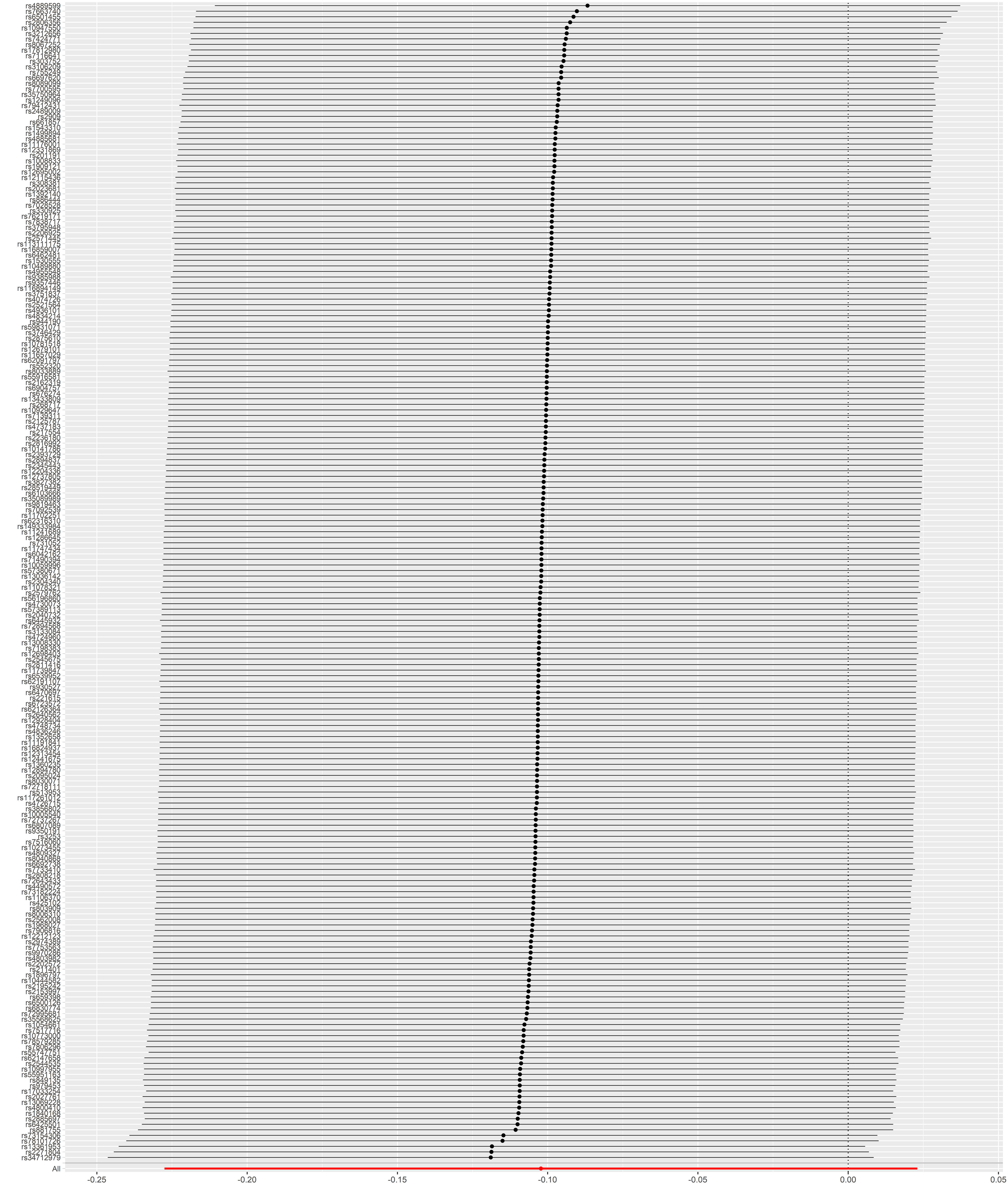

Figure S10. A. MR leave-one-out sensitivity analysis for FEV1 on VTE

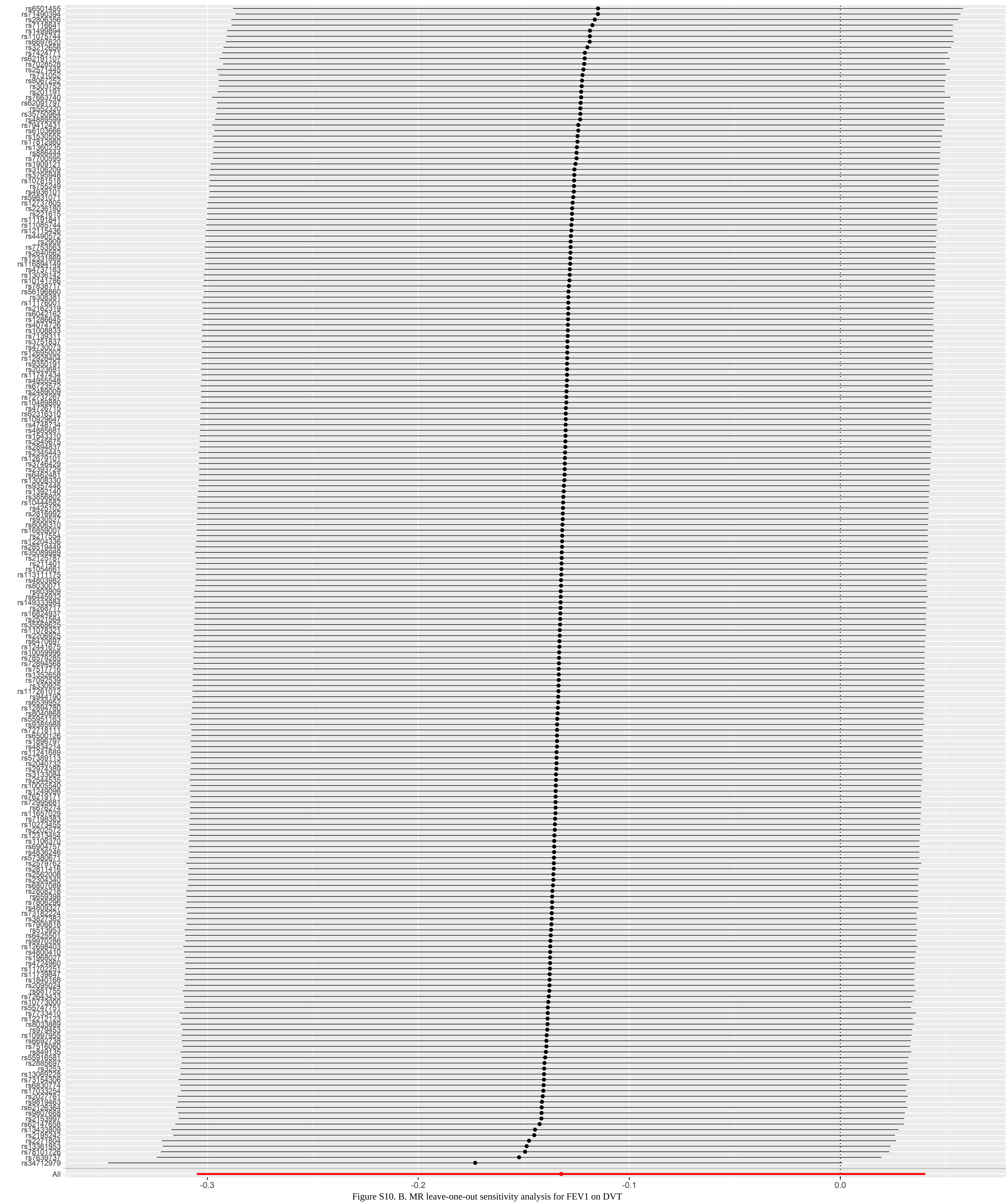

Figure S10. B. MR leave-one-out sensitivity analysis for FEV1 on DVT

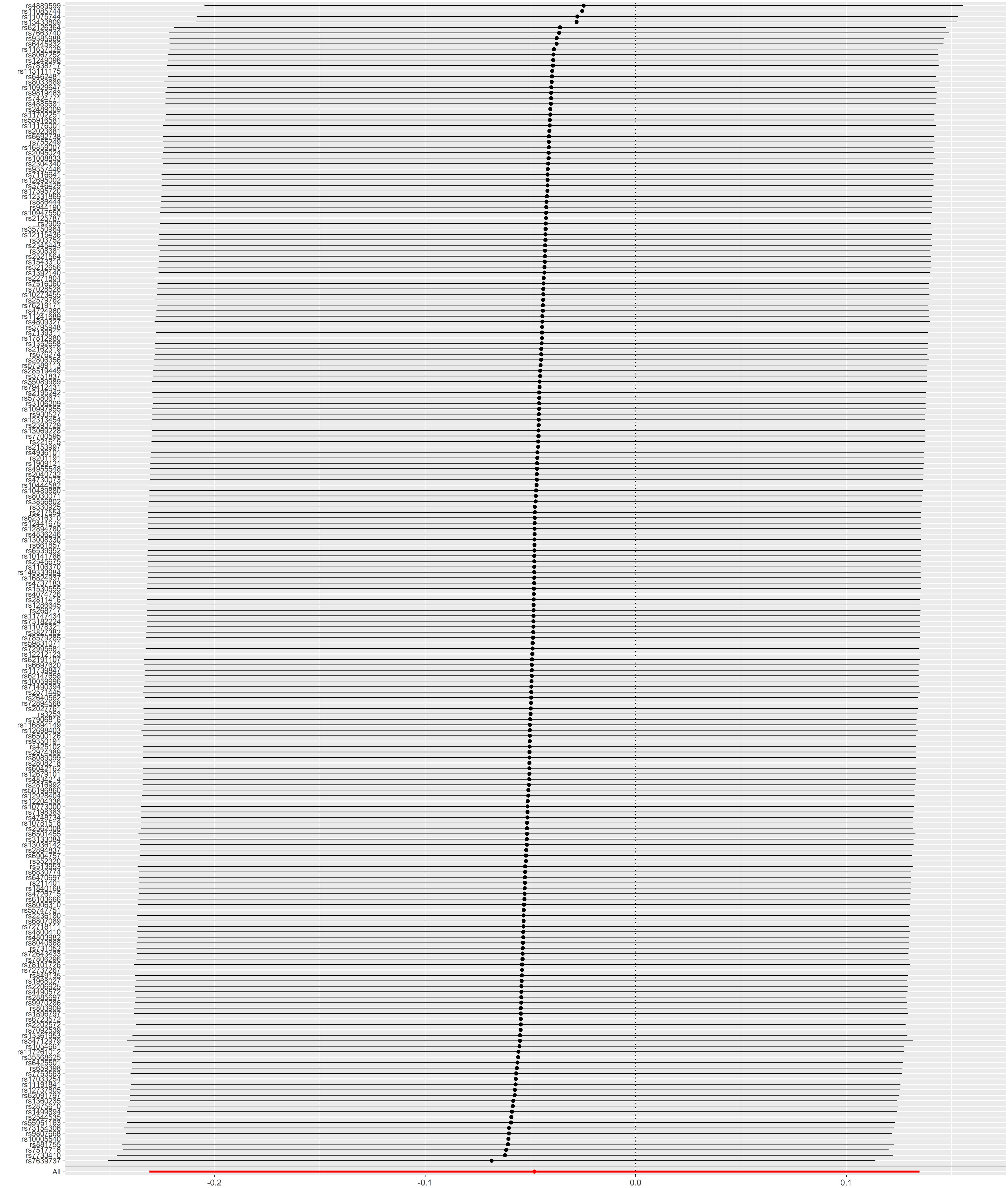

Figure S10. C. MR leave-one-out sensitivity analysis for FEV1 on PE

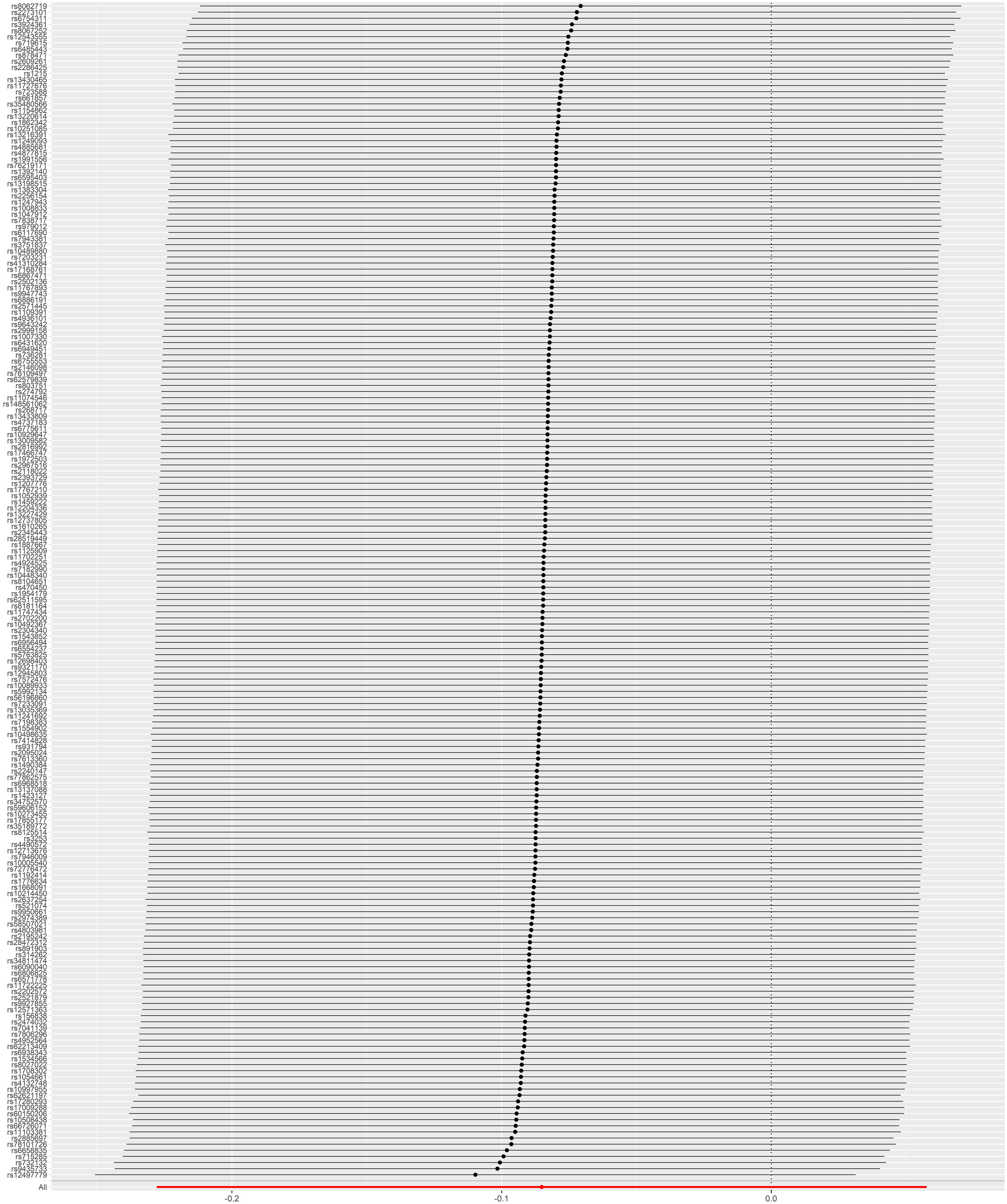

Figure S11. A. MR leave-one-out sensitivity analysis for FVC on VTE

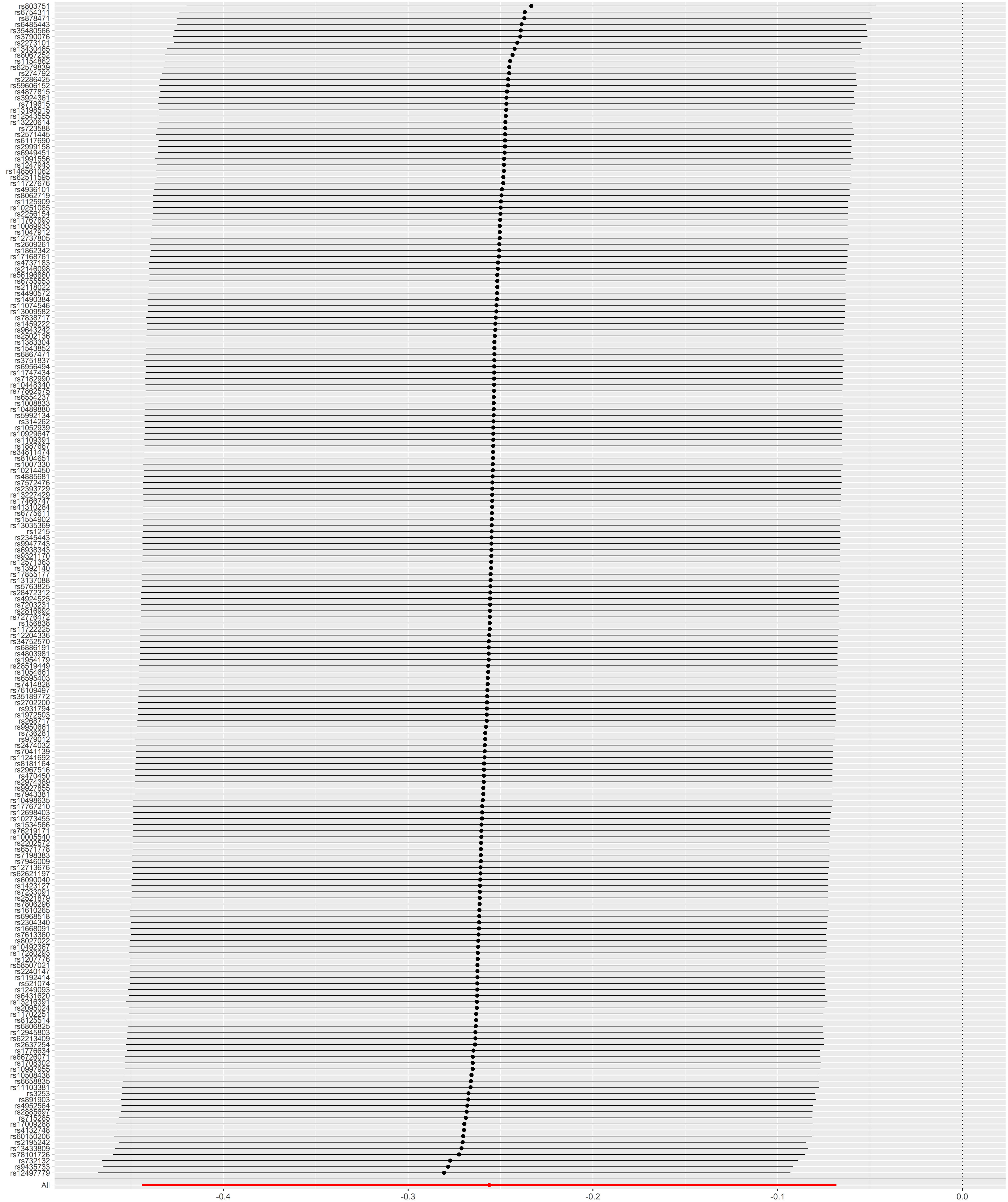

Figure S11. B. MR leave-one-out sensitivity analysis for FVC on DVT

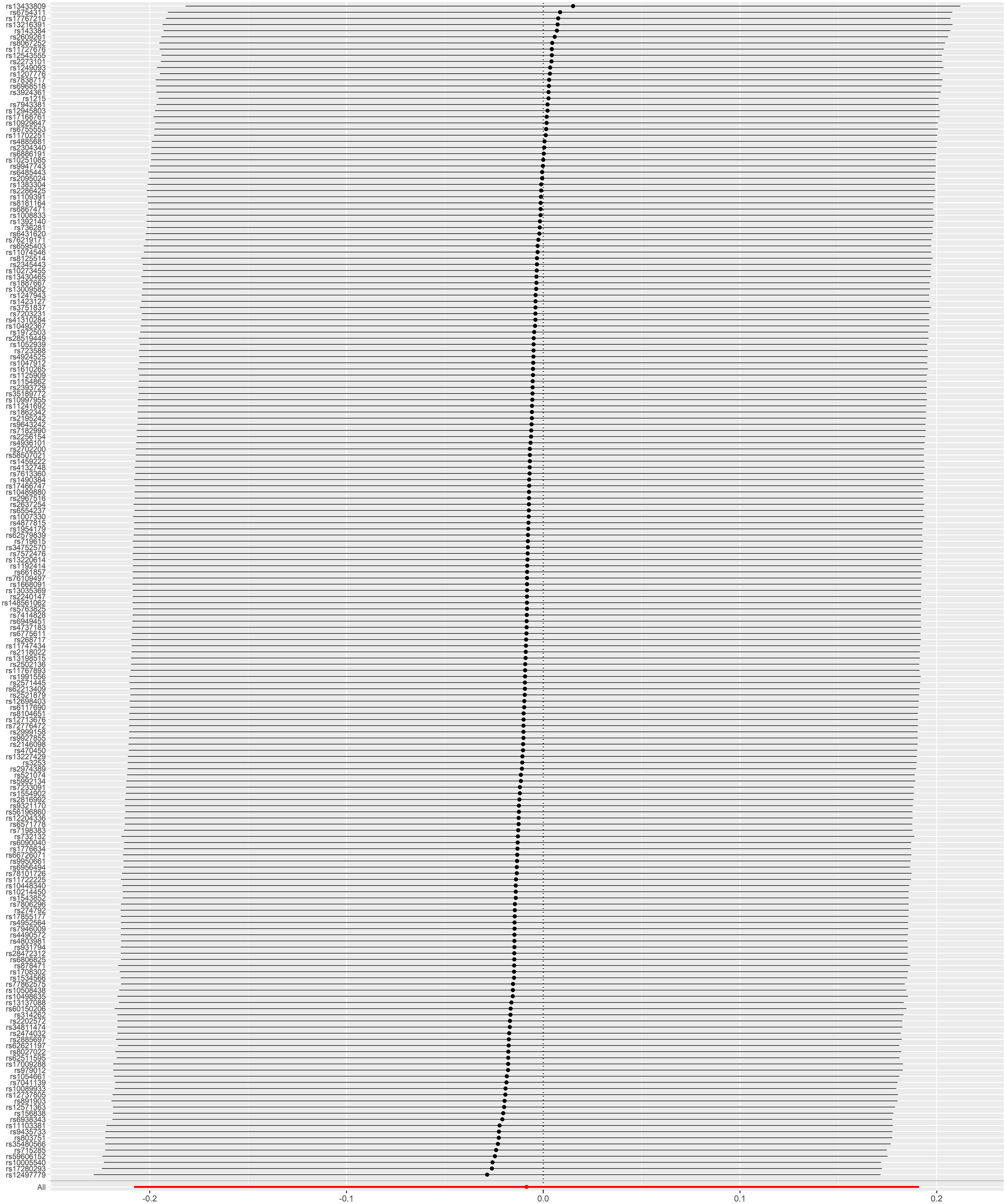

Figure S11. C. MR leave-one-out sensitivity analysis for FVC on PE
