# Supplementary material for: Genetic association and causal inference between lung function and venous thromboembolism
Source: Respir Res. 2023 Jan 30;24:36. doi: 10.1186/s12931-023-02335-3 (PMC9885683; doi:10.1186/s12931-023-02335-3)
Supplement: Supplementary file 4 — Additional file 4: Figure S12. A MR leave-one-out sensitivity analysis for FEV1/FVC on VTE. B MR leave-one-out sensitivity analysis for FEV1/FVC on DVT. C MR leave-one-out sensitivity analysis for FEV1/FVC on PE. Figure S13. A MR leave-one-out sensitivity analysis for PEF on VTE. B MR leave-one-out sensitivity analysis for PEF on DVT. C MR leave-one-out sensitivity analysis for PEF on PE. [file 12931_2023_2335_MOESM4_ESM.pdf]

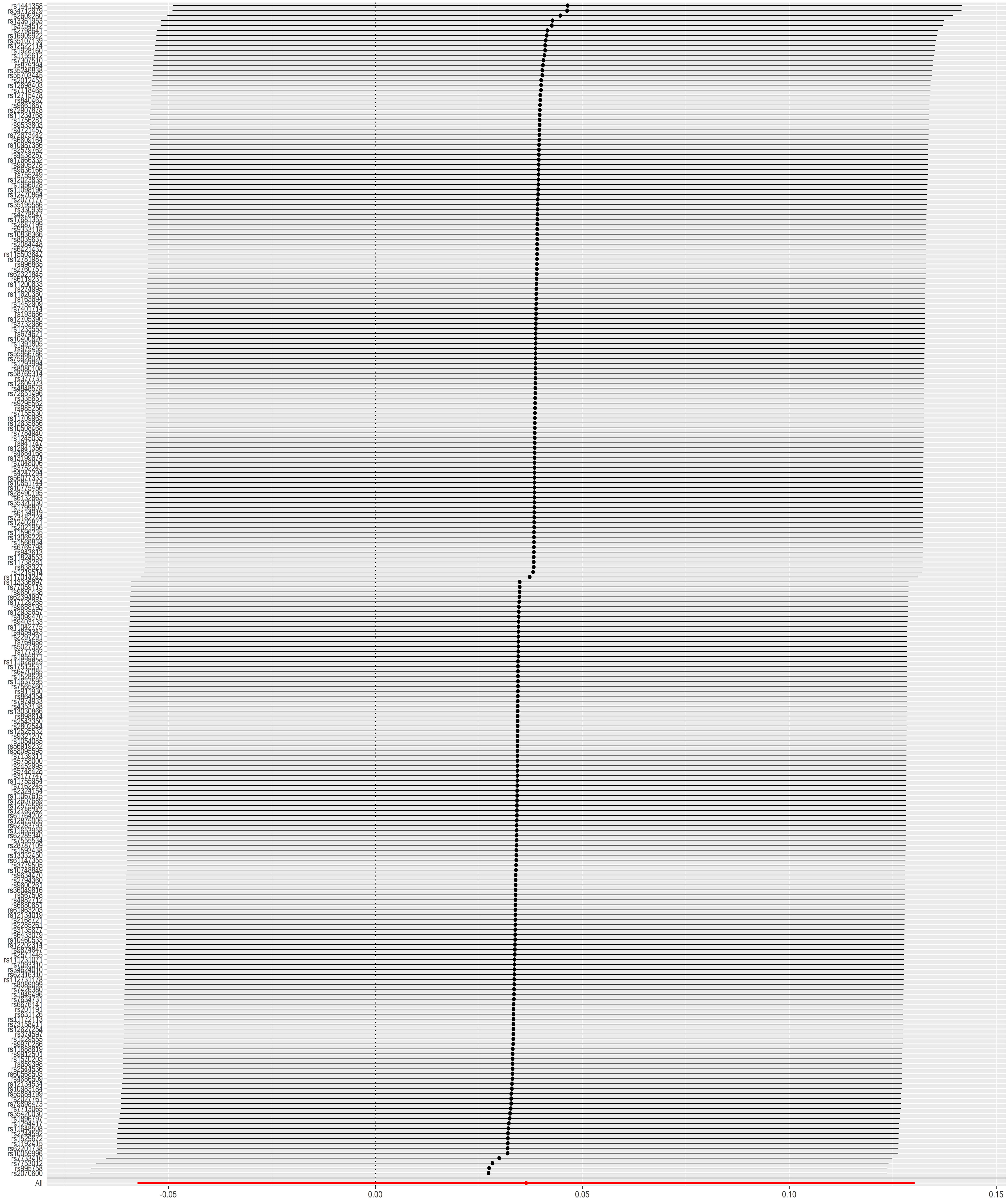

Figure S12. A. MR leave-one-out sensitivity analysis for FEV1/FVC on VTE

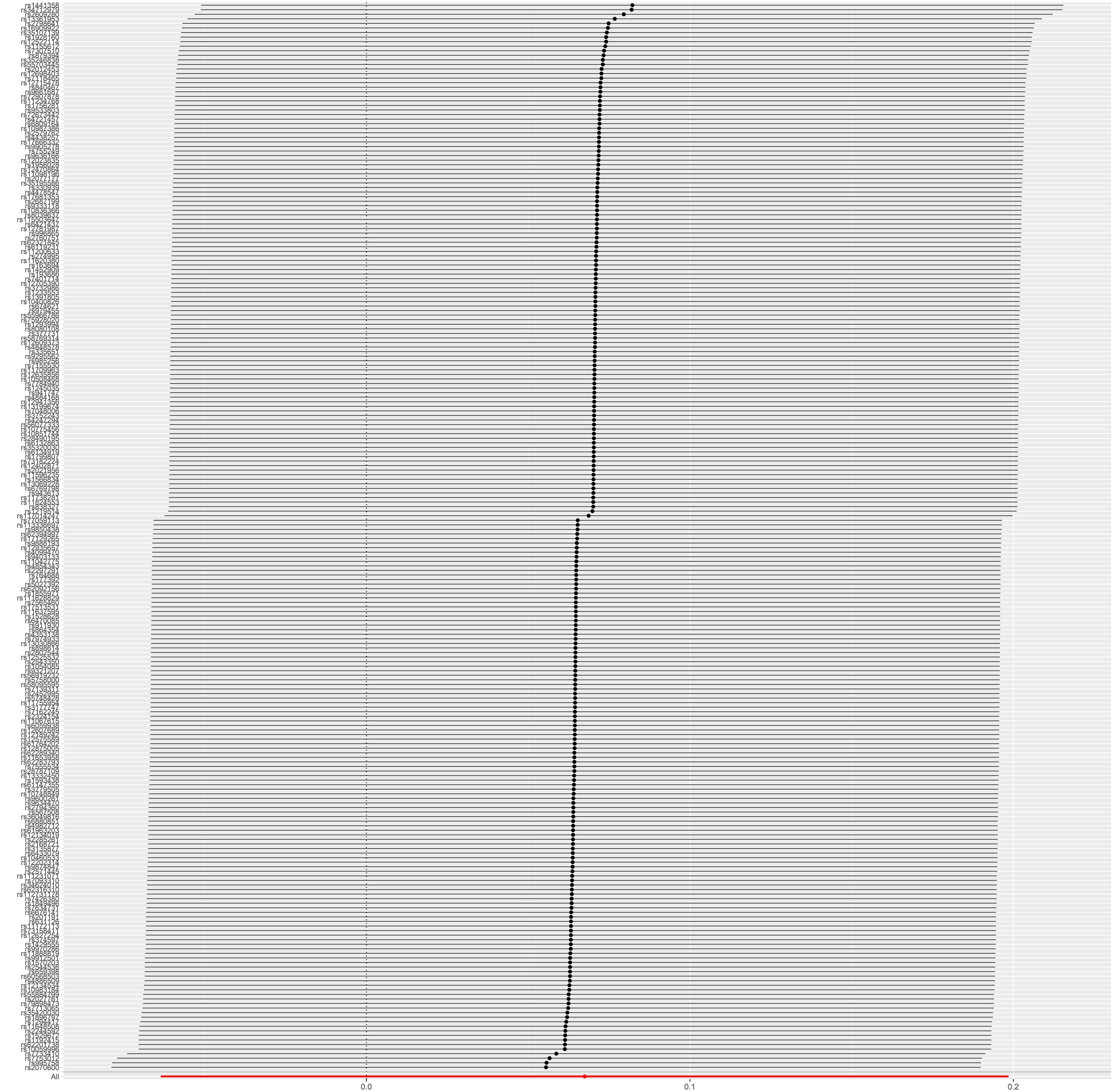

Figure S12. B. MR leave-one-out sensitivity analysis for FEV1/FVC on DVT

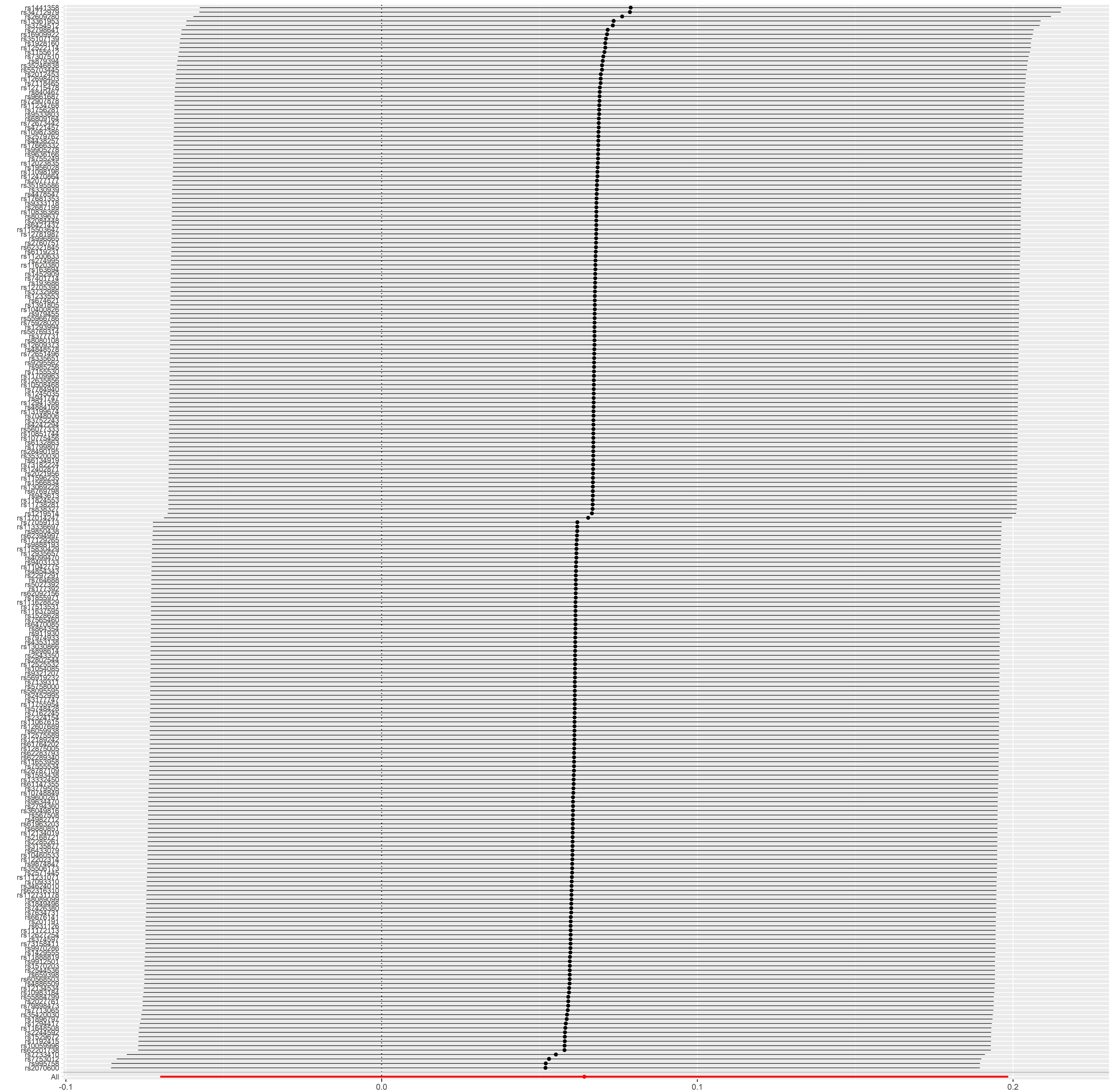

Figure S12. C. MR leave-one-out sensitivity analysis for FEV1/FVC on PE

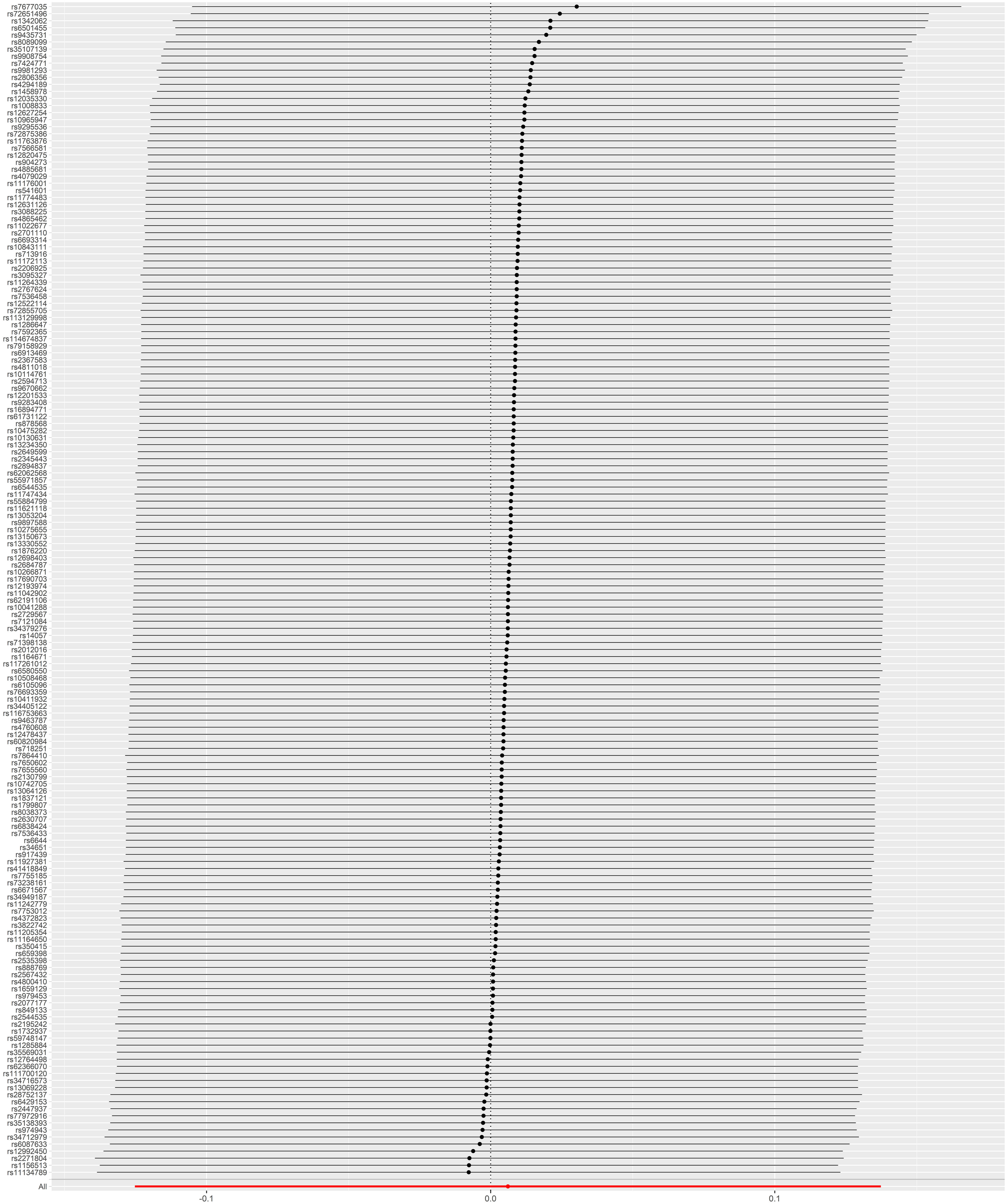

Figure S13. A. MR leave-one-out sensitivity analysis for PEF on VTE

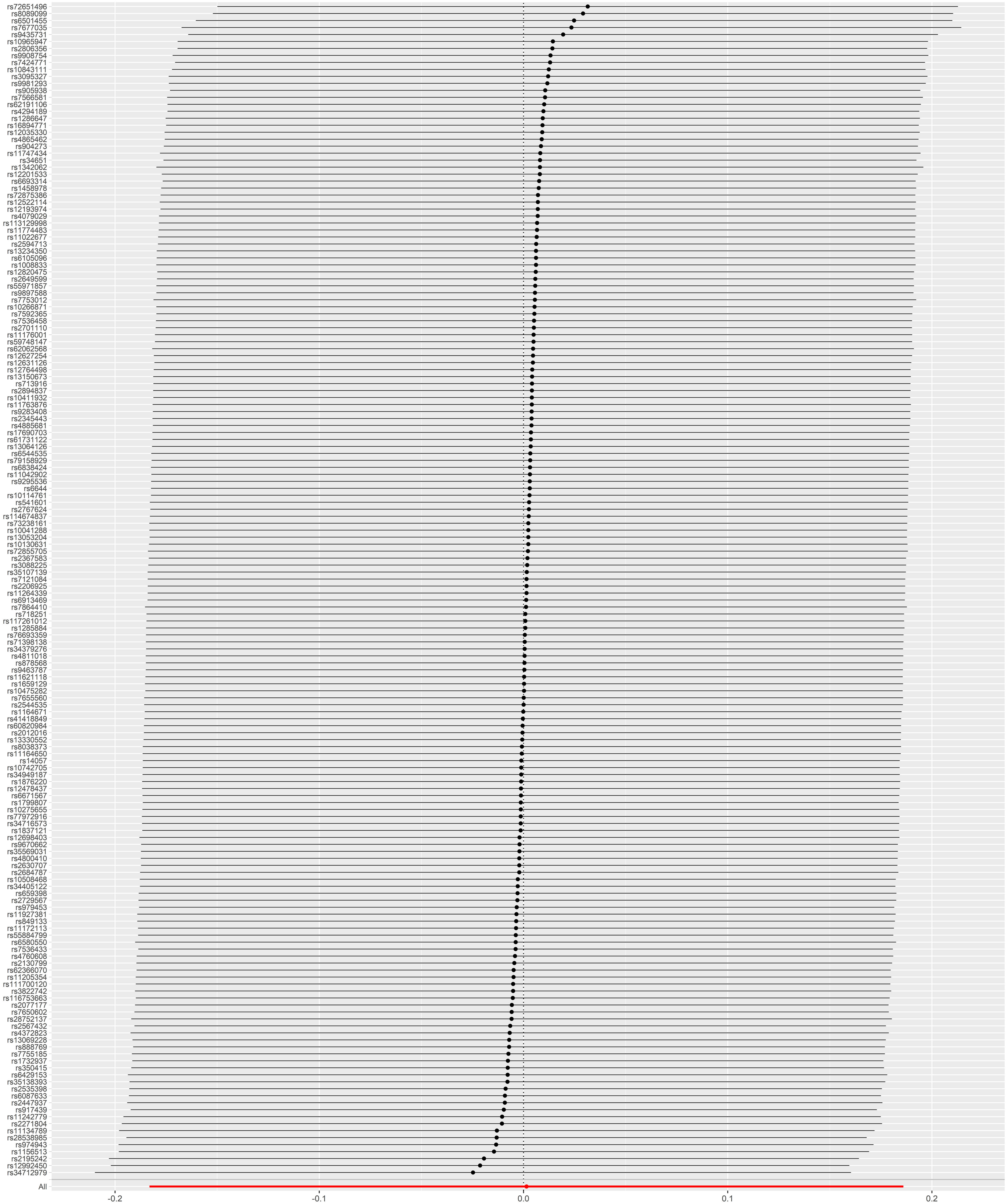

Figure S13. B. MR leave-one-out sensitivity analysis for PEF on DVT

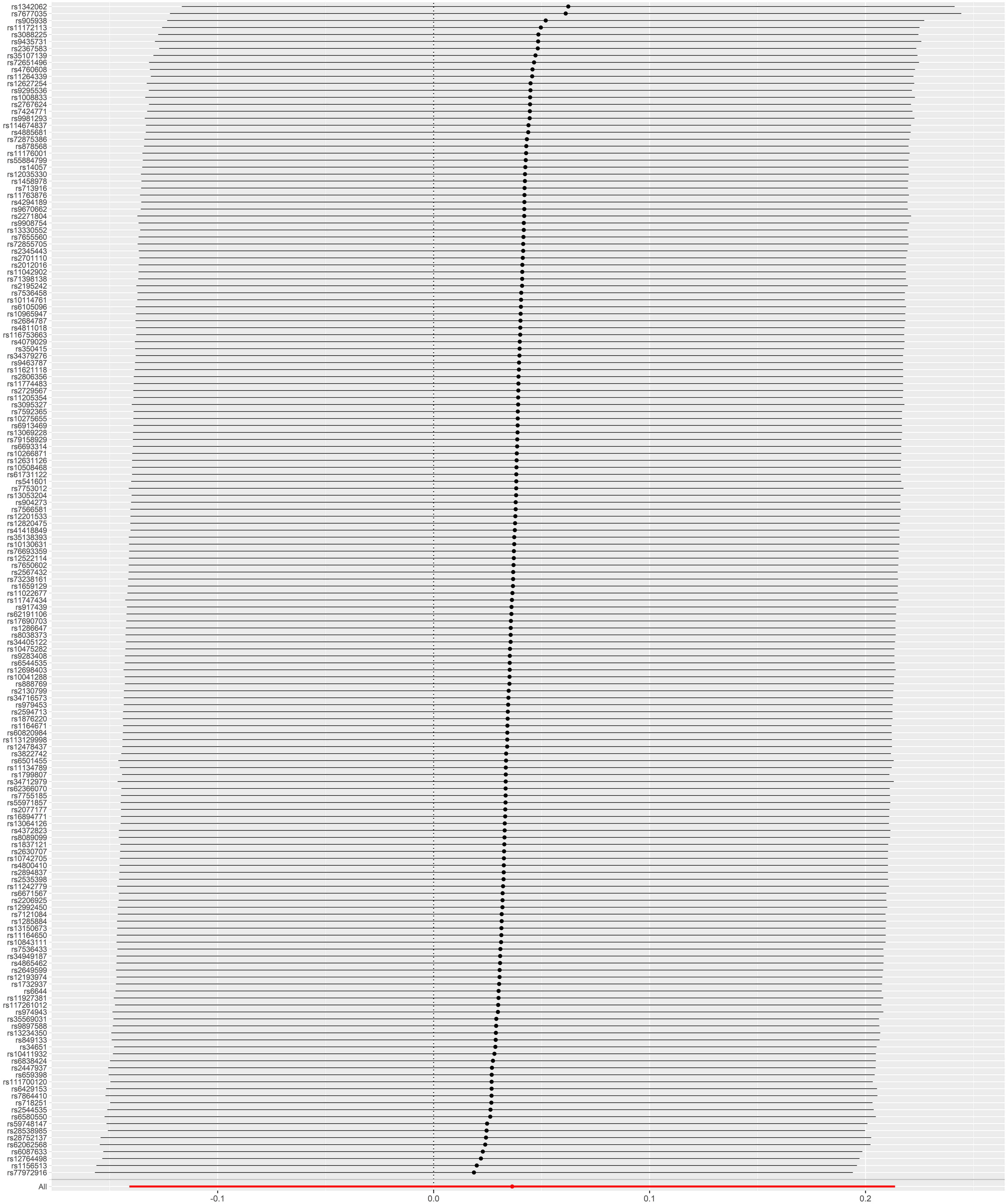

Figure S13. C. MR leave-one-out sensitivity analysis for PEF on PE
