# Supplementary material for: Genetic association and causal inference between lung function and venous thromboembolism
Source: Respir Res. 2023 Jan 30;24:36. doi: 10.1186/s12931-023-02335-3 (PMC9885683; doi:10.1186/s12931-023-02335-3)
Supplement: Supplementary file 5 — Additional file 5: Figure S14. A MR leave-one-out sensitivity analysis for VTE on FEV1. B MR leave-one-out sensitivity analysis for VTE on FVC. C MR leave-one-out sensitivity analysis for VTE on FEV1/FVC. D MR leave-one-out sensitivity analysis for VTE on PEF. Figure S15. A MR leave-one-out sensitivity analysis for DVT on FEV1. B MR leave-one-out sensitivity analysis for DVT on FVC. C MR leave-one-out sensitivity analysis for DVT on FEV1/FVC. D MR leave-one-out sensitivity analysis for DVT on PEF. Figure S16. A MR leave-one-out sensitivity analysis for PE on FEV1. B MR leave-one-out sensitivity analysis for PE on FVC. C MR leave-one-out sensitivity analysis for PE on FEV1/FVC. D MR leave-one-out sensitivity analysis for PE on PEF. [file 12931_2023_2335_MOESM5_ESM.pdf]

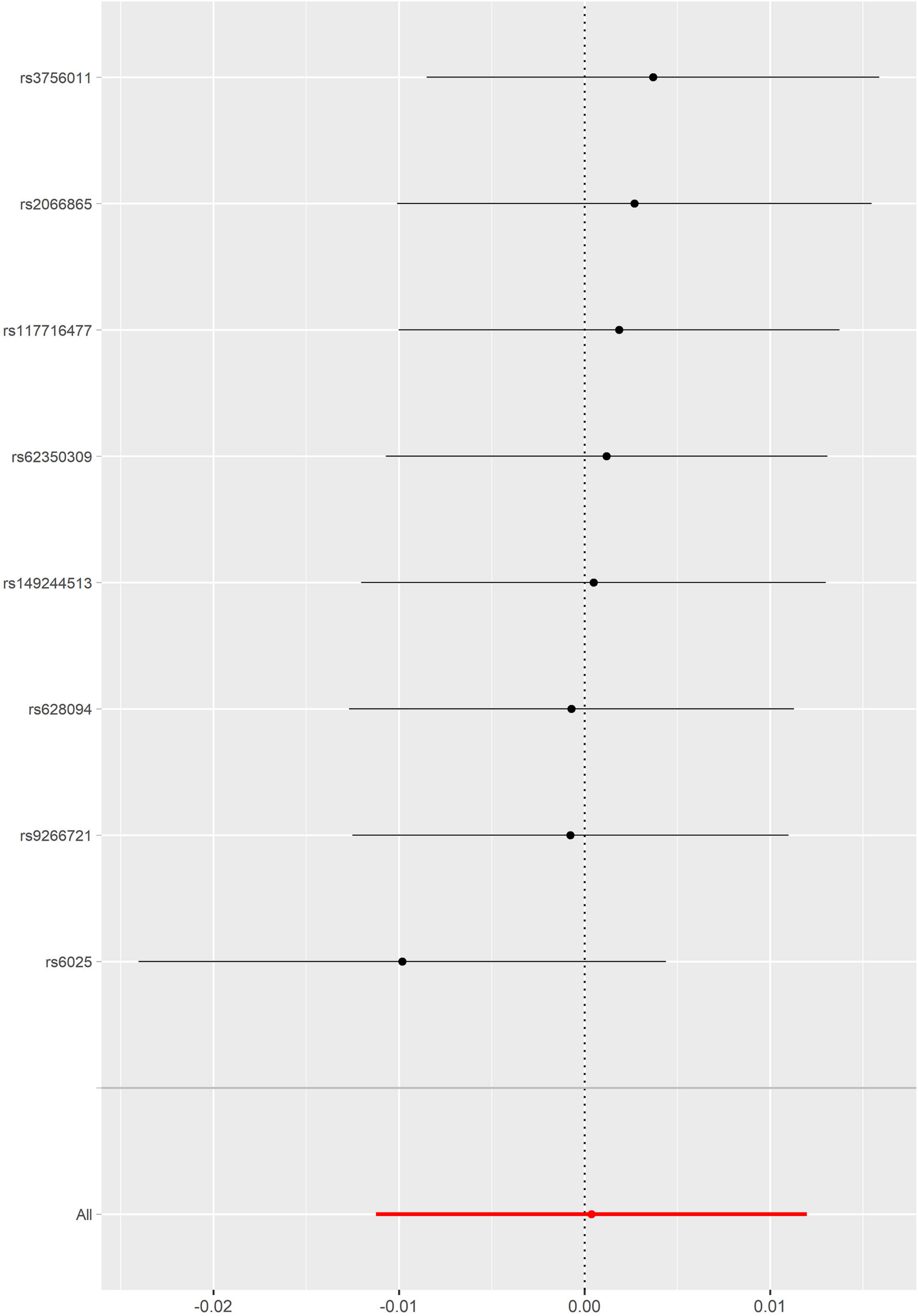

Figure S14. A. MR leave-one-out sensitivity analysis for VTE on FEV1

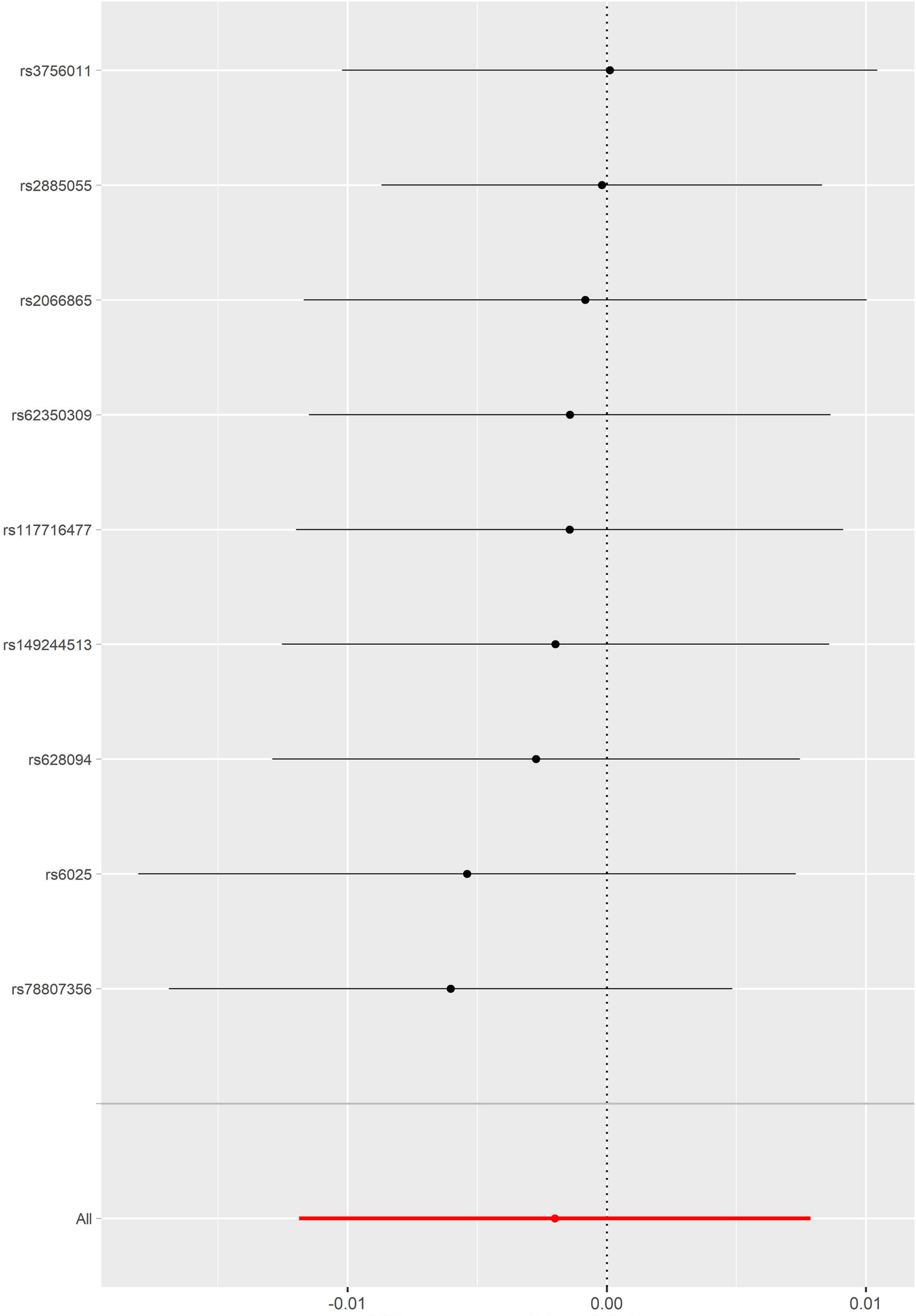

Figure S14. B. MR leave-one-out sensitivity analysis for VTE on FVC

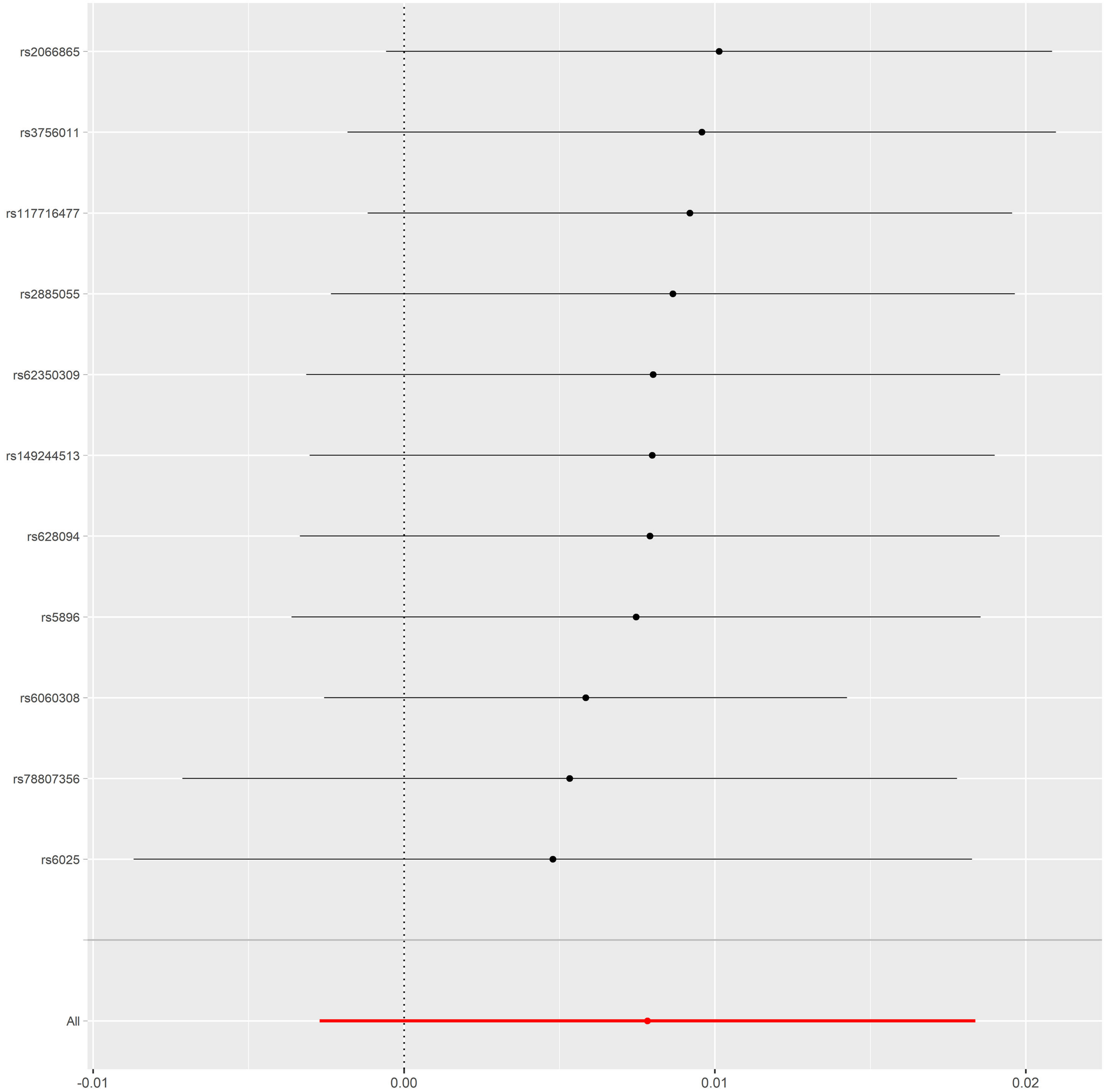

Figure S14. C. MR leave-one-out sensitivity analysis for VTE on FEV1/FVC

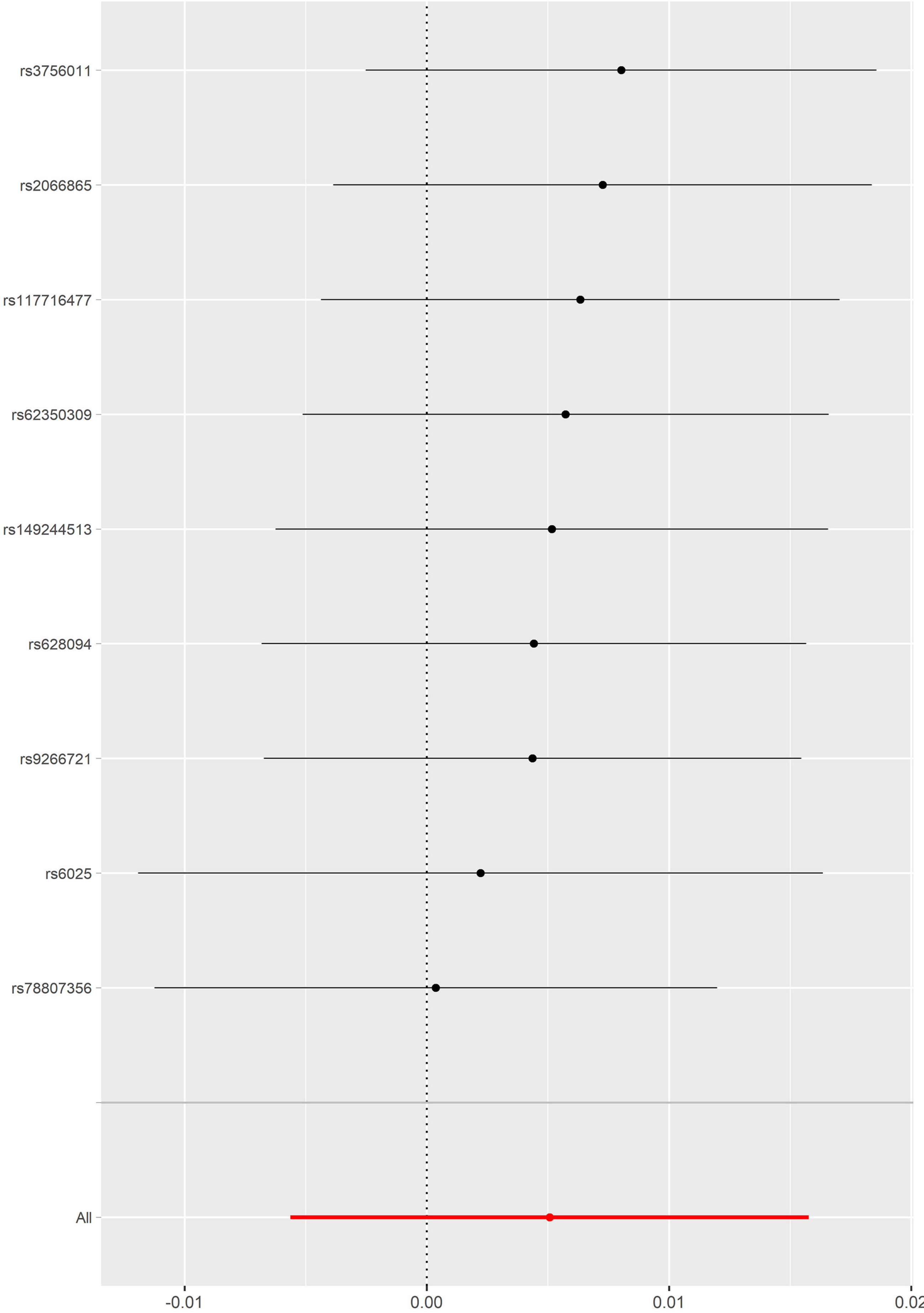

Figure S14. D. MR leave-one-out sensitivity analysis for VTE on PEF

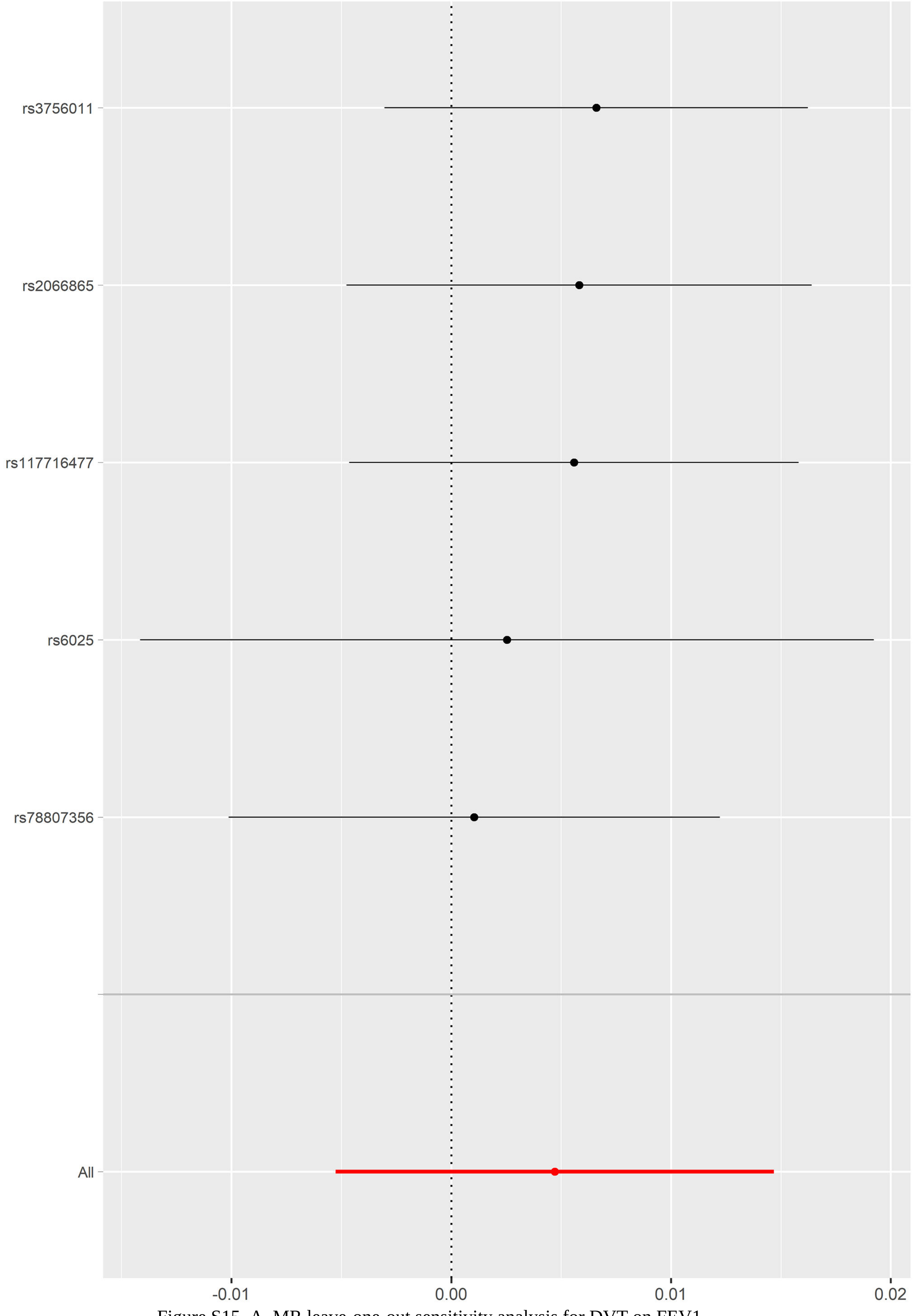

Figure S15. A. MR leave-one-out sensitivity analysis for DVT on FEV1

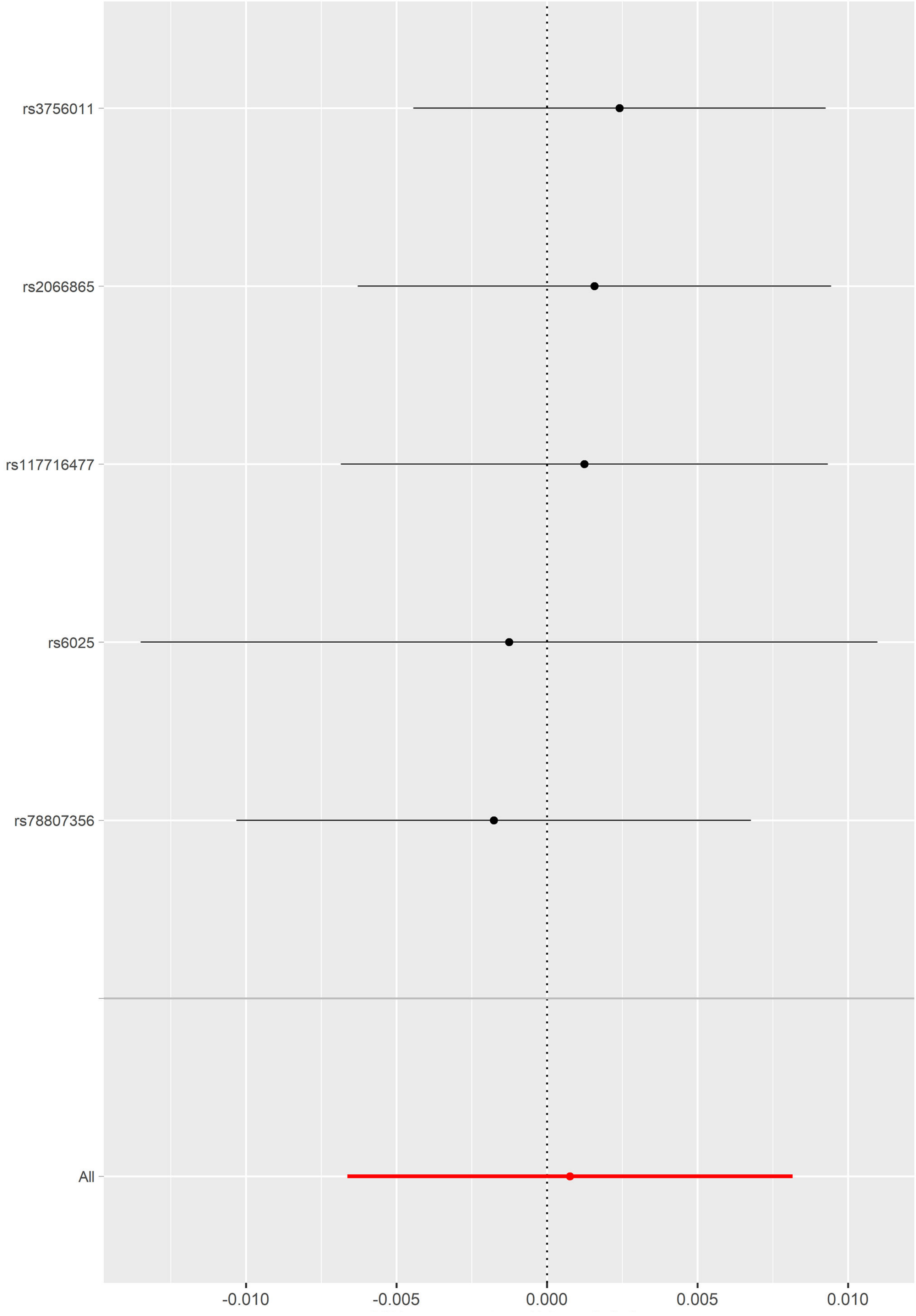

Figure S15. B. MR leave-one-out sensitivity analysis for DVT on FVC

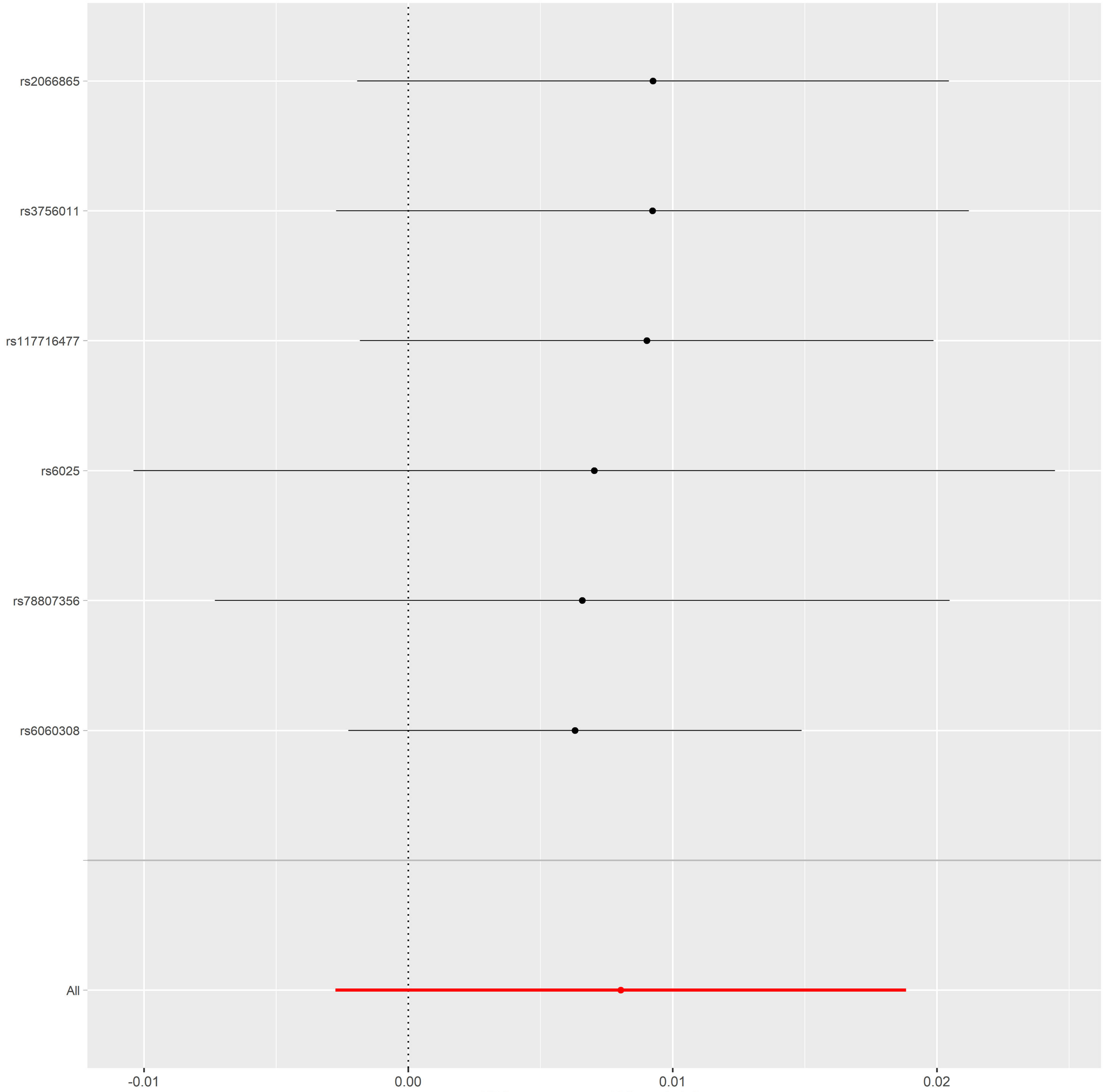

Figure S15. C. MR leave-one-out sensitivity analysis for DVT on FEV1/FVC

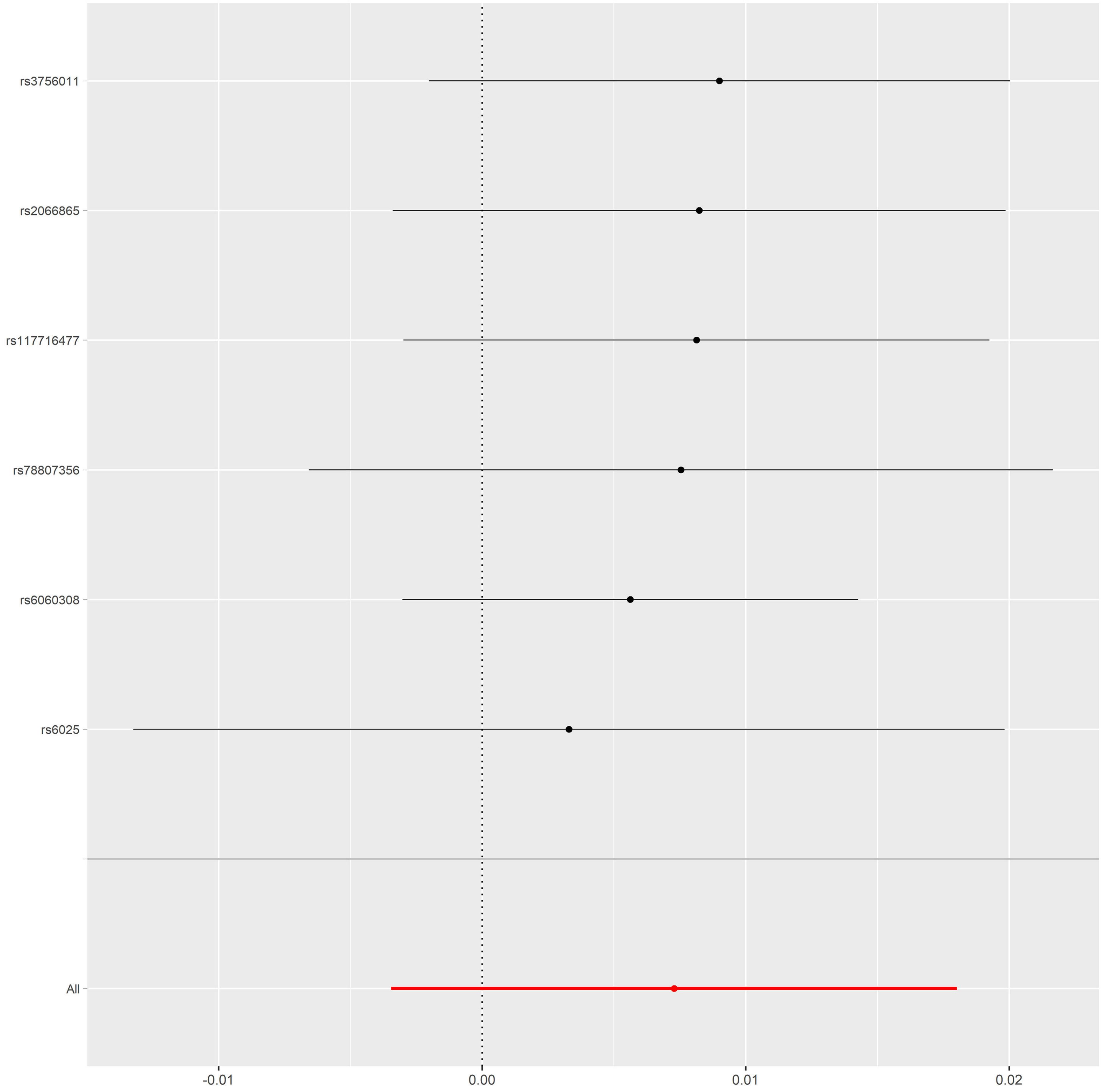

Figure S15. D. MR leave-one-out sensitivity analysis for DVT on PEF

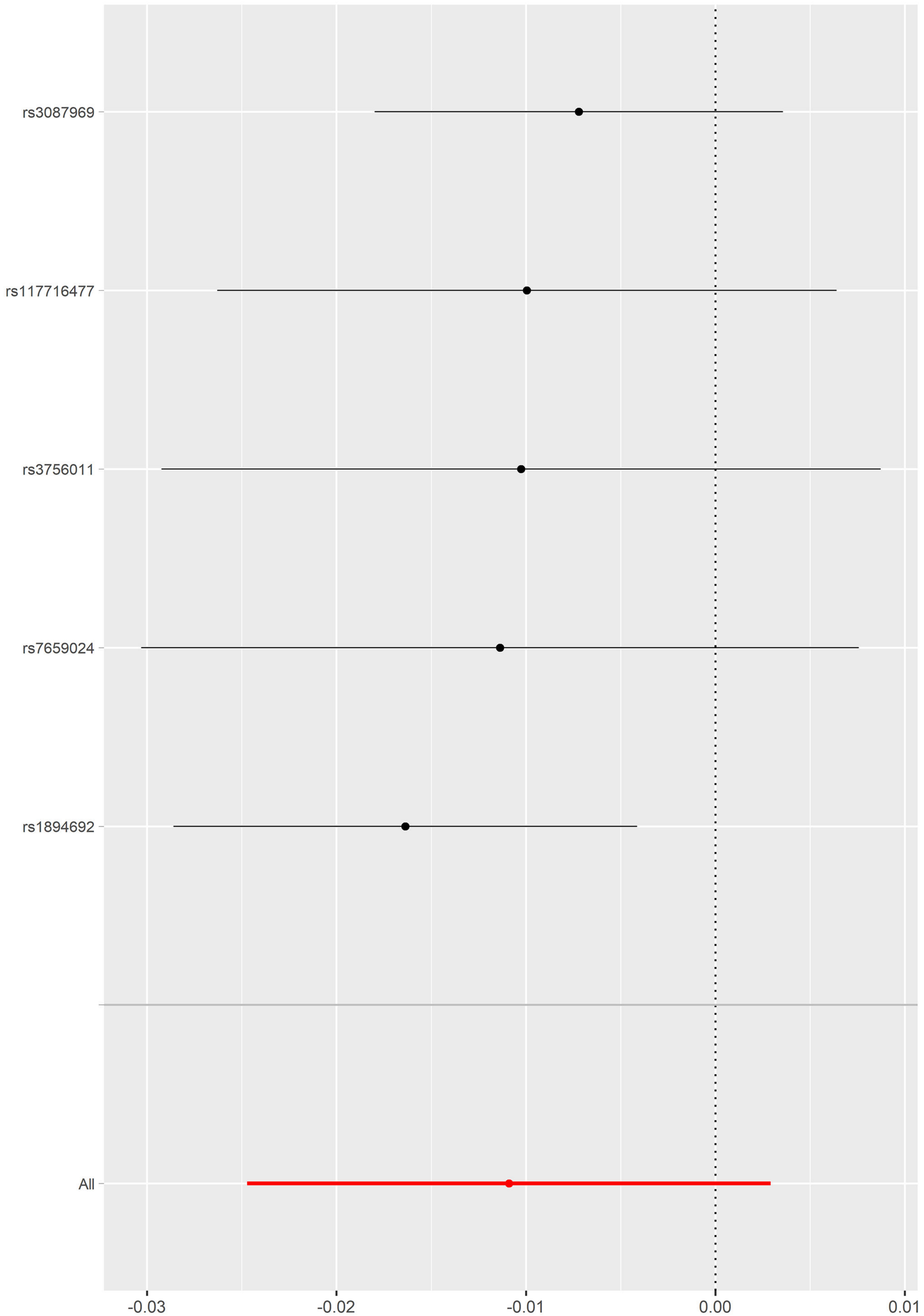

Figure S16. A. MR leave-one-out sensitivity analysis for PE on FEV1

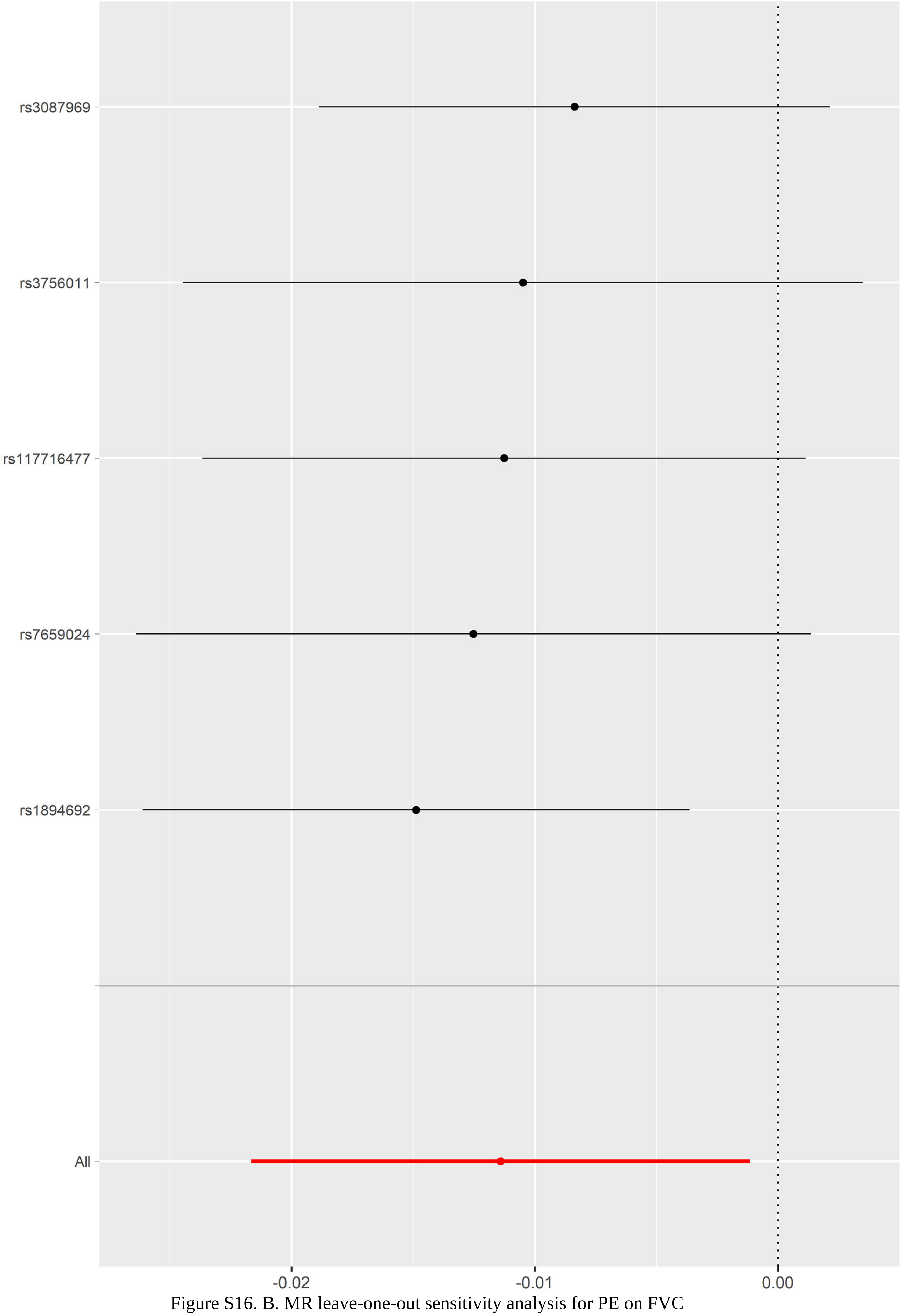

Figure S16. B. MR leave-one-out sensitivity analysis for PE on FVC

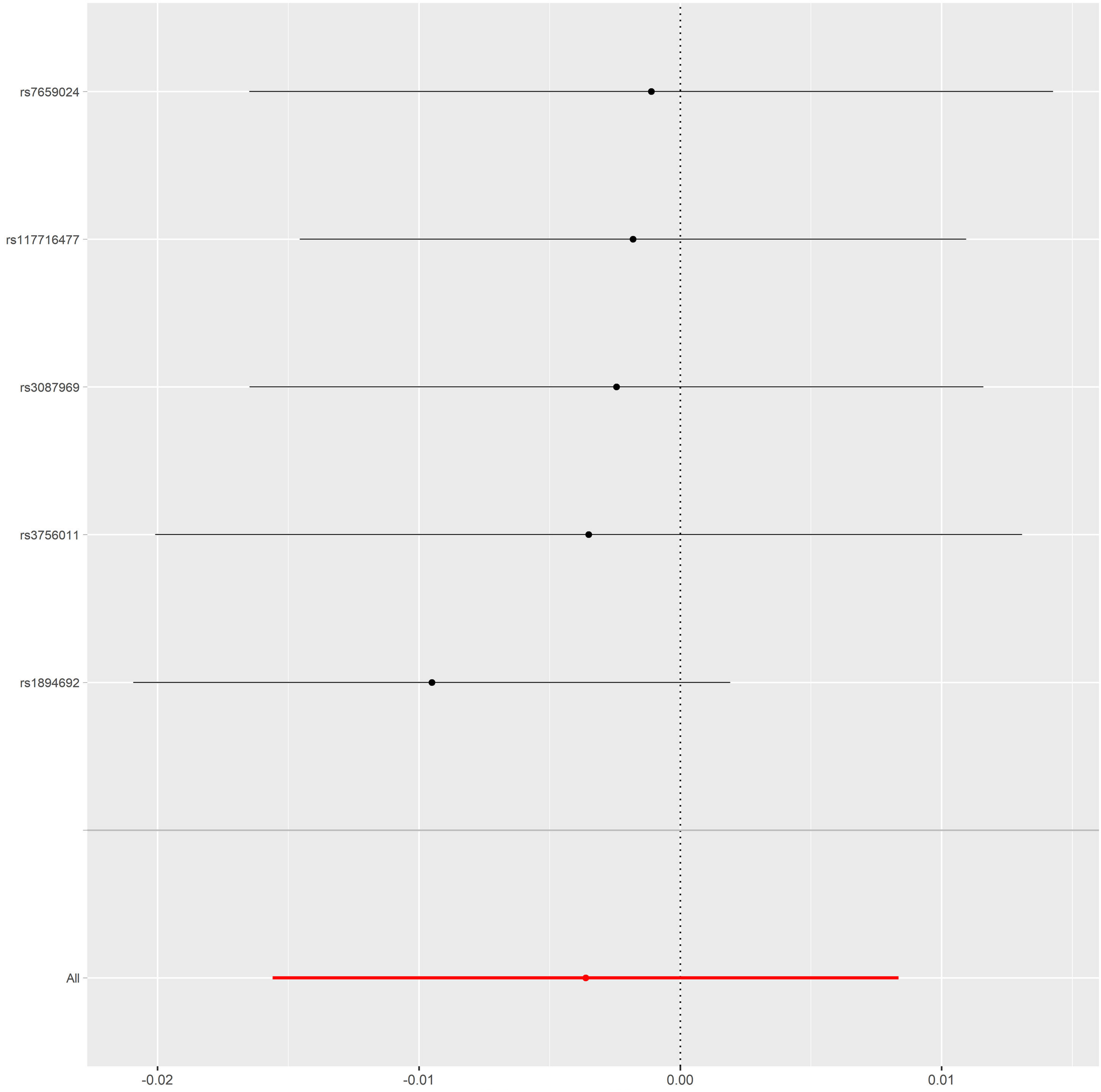

Figure S16. C. MR leave-one-out sensitivity analysis for PE on FEV1/FVC

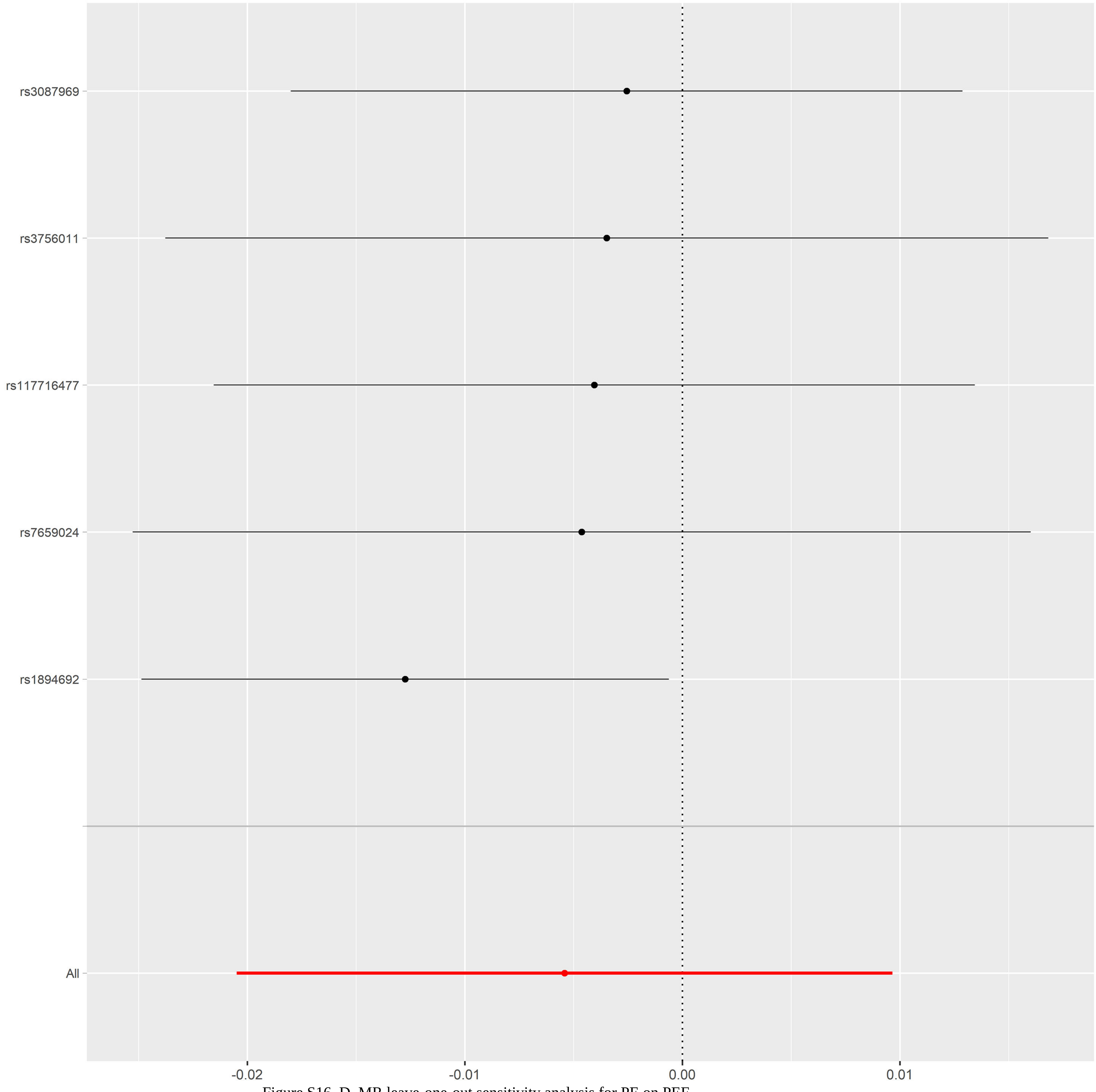

Figure S16. D. MR leave-one-out sensitivity analysis for PE on PEF
